# Proteomic Investigations of Autism Brain Identify Known and Novel Pathogenetic Processes

**DOI:** 10.1038/s41598-019-49533-y

**Published:** 2019-09-11

**Authors:** Joseph R. Abraham, Nicholas Szoko, John Barnard, Robert A. Rubin, Daniela Schlatzer, Kathleen Lundberg, Xiaolin Li, Marvin R. Natowicz

**Affiliations:** 1 Cleveland Clinic Lerner College of Medicine of Case Western Reserve University, Cleveland, OH 44195 USA; 2 Department of Quantitative Health Sciences, Lerner Research Institute, Cleveland Clinic, Cleveland, OH 44195 USA; 3 Department of Mathematics, Whittier College, Whittier, CA 90602 USA; 4 Center for Proteomics, Case Western Reserve University, Cleveland, OH 44106 USA; 5 Pathology and Laboratory Medicine, Genomic Medicine, Neurological and Pediatrics Institutes, Cleveland Clinic, Cleveland, OH 44195 USA

**Keywords:** Autism spectrum disorders, Autism spectrum disorders, Molecular medicine, Autism spectrum disorders, Autism spectrum disorders

## Abstract

Autism Spectrum Disorder (ASD) is a set of heterogeneous neurodevelopmental conditions defined by impairments in social communication and restricted, repetitive behaviors, interests or activities. Only a minority of ASD cases are determined to have a definitive etiology and the pathogenesis of most ASD is poorly understood. We hypothesized that a global analysis of the proteomes of human ASD vs. control brain, heretofore not done, would provide important data with which to better understand the underlying neurobiology of autism. In this study, we characterized the proteomes of two brain regions, Brodmann area 19 (BA19) and posterior inferior cerebellum (CB), from carefully selected idiopathic ASD cases and matched controls using label-free HPLC-tandem mass spectrometry. The data revealed marked differences between ASD and control brain proteomes for both brain regions. Unlike earlier transcriptomic analyses using frontal and temporal cortex, however, our proteomic analysis did not support ASD attenuating regional gene expression differences. Bioinformatic analyses of the differentially expressed proteins between cases and controls highlighted canonical pathways involving glutamate receptor signaling and glutathione-mediated detoxification in both BA19 and CB; other pathways such as Sertoli cell signaling and fatty acid oxidation were specifically enriched in BA19 or CB, respectively. Network analysis of both regions of ASD brain showed up-regulation of multiple pre- and post-synaptic membrane or scaffolding proteins including glutamatergic ion channels and related proteins, up-regulation of proteins involved in intracellular calcium signaling, and down-regulation of neurofilament proteins, with DLG4 and MAPT as major hub proteins in BA19 and CB protein interaction networks, respectively. Upstream regulator analysis suggests neurodegeneration-associated proteins drive the differential protein expression for ASD in both BA19 and CB. Overall, the proteomic data provide support for shared dysregulated pathways and upstream regulators for two brain regions in human ASD brain, suggesting a common ASD pathophysiology that has distinctive regional expression.

## Introduction

Autism Spectrum Disorder (ASD) is a clinically heterogeneous group of neurodevelopmental conditions characterized by early age-of-onset impairments in social communication and social interaction and restricted, repetitive patterns of behaviors, interests or activities^1,2^. Persons with ASD commonly also have medical, neurological and/or psychiatric co-morbidities^3,4^ and, as ASD is a lifelong condition, there are many years lived with disability^5^. Consequently, ASD is associated with varied and considerable personal, family and societal challenges and costs^6^. ASD is also common, with an estimated global prevalence of about 1–1.5%^7,8^. For these reasons, determination of the underlying pathobiology of ASD and efforts directed toward ASD prevention, early diagnosis, and effective treatment are public health priorities.

More than 100 mendelian and cytogenomic variants have been causally associated with ASD, all of which are uncommon or rare and many of which are incompletely characterized^9–11^; environmental factors, while clearly relevant, are even less well understood^12^. At this time, despite extensive diagnostic evaluation, the vast majority of persons with ASD do not receive an etiological diagnosis and receive a diagnosis of autism of unknown etiology or idiopathic autism; only about 10–25% of cases have defined pathogenic abnormalities that are determinative or significantly contributory to ASD causation^10^. The marked etiologic heterogeneity of ASD, the diversity of known and purported etiologies, and the predominance of idiopathic cases underscore the complexity and challenge inherent in uncovering the underlying pathogenesis of ASD^13^.

Diverse investigative approaches - from neuropathological evaluation to functional neuroimaging - suggest that persons with ASD have central nervous system (CNS) developmental dysconnectivity, and important theories of ASD pathogenesis center on abnormal synaptic homeostasis and the abnormal development or regulation of neuronal circuitry^14–16^. Considerable efforts to unravel the mechanisms underlying the developmental dysconnectivity of ASD are ongoing. Molecular analyses have revealed a wide array of contributors to these processes including genes and proteins involved in diverse cellular processes including synapse biology, regulation of neuronal gene transcription and translation, Wnt signaling and mitochondrial bioenergetics, among others^10,17–21^. There is, however, a notable gap in the pathophysiological characterization of ASD at the level of the proteome.

Proteomics, the large-scale study of protein expression in cells or tissues, enables an analysis of protein expression profiles and the determination of biologically relevant protein interaction networks and signaling pathways^22^. Different proteomic methodologies and experimental designs can be used for diverse purposes, from providing an unbiased relative quantification of a large number of proteins expressed in cells or tissues, often designated as label-free expression proteomics, to the quantification of particular targeted proteins or isoforms of proteins, or groups of proteins having a specific post-translational modification^23^. Proteomic analysis affords a potentially complementary approach to genetic analyses in characterizing the pathophysiology of a phenotype and has an advantage in being ‘closer’ to the expression of the cellular or tissue phenotype than studies done at a genomic or transcriptomic level.

Despite the power of proteomics and its increasing utilization in the understanding of neurological and developmental disorders, there are few applications of proteomic approaches to the study of ASD^24,25^. Moreover, most of the proteomic studies of ASD have examined non-neural tissue and no consistent findings have been apparent^25^.

In this study, we sought to perform a global label-free proteomic analysis of stringently selected idiopathic ASD post-mortem brain samples and carefully matched controls as a means to better understand ASD pathobiology. Two brain regions were evaluated in cases and controls: Brodmann area 19 region of occipital cortex (BA19) and the left posterior inferior cerebellar hemispheric cortex (CB); both regions are affected in ASD and have been studied extensively^26–29^. We hypothesized that brain proteomic analysis would show distinctive protein expression profiles between the two brain regions and also between ASD and controls for these regions and that analyses of these differences would provide insights regarding the underlying biology of ASD.

## Results

### The proteomes of BA19 and CB are distinct from each other and between ASD cases and controls

**Figure 1:**
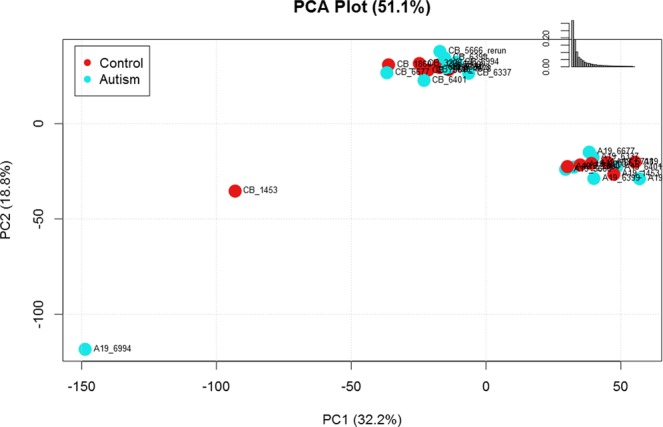
Principal component analysis of peptide expression data showing clear separation of samples by brain region and one outlier (BA19_6994).

The proteomic analysis of ASD and control samples detected 9205 peptides belonging to 1807 proteins in BA19 occipital cortex and 8017 peptides from 1640 proteins in cerebellar (CB) tissue. For BA19, 63% of proteins were identified by more than one uniquely identifying peptide; similarly, 64% of CB proteins were identified by more than one peptide. Principal component analysis, based on the peptides noted in each brain region, revealed complete separation of the two regions, indicating the differences in protein expression between BA19 and cerebellum (Fig. 1).

Analysis of proteins identified by at least 2 unique peptides revealed 146 proteins from BA19 having significant differential expression between ASD and controls (p < 0.05) with 27 remaining significant after false discovery rate (FDR) adjustment (Benjamini-Hochberg (BH) adjusted p < 0.05); 191 proteins were found to be significantly differentially expressed in CB (p < 0.05) with 38 proteins remaining significant after FDR adjustment (BH adjusted p < 0.05). Supplementary Table 1 provides subject characteristics and Supplementary Table 2 notes all differentially expressed proteins identified by ≥2 peptides from each brain region with p-values, BH adjusted p-values, and associated percent expression changes.

### Brain regional protein differences are maintained in ASD

**Figure 2:**
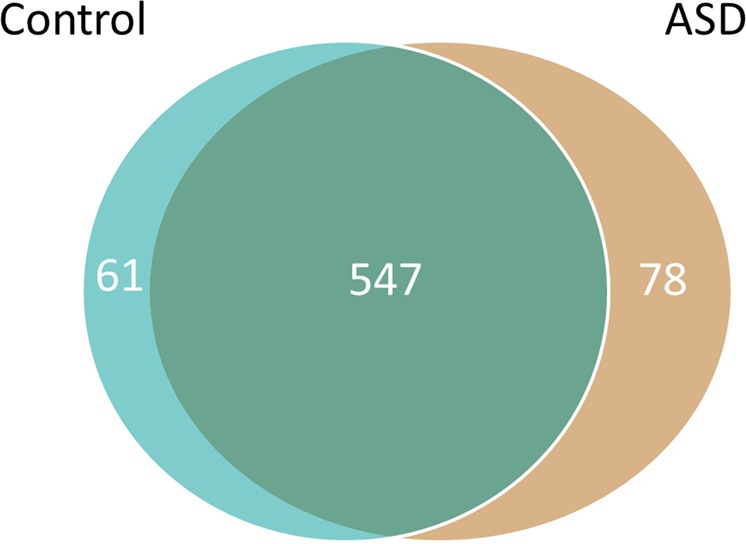
Differentially expressed proteins between cerebellar cortex and occipital cortex (BA19) comparing ASD and control brains. The differentially expressed proteins common to both types of brain as well as numbers unique to each are noted.

Previous transcriptomic work showed an attenuation of normal gene expression differences between frontal and temporal cortex in ASD compared to control brains^30,31^. Analysis of proteins differentially expressed (p < 0.05) by brain region in our ASD and control data sets showed 625 differentially expressed proteins noted between the BA19 and CB regions in the ASD brains and 608 differentially expressed proteins noted between the BA19 and CB regions in the control brains. The vast majority of these differentially expressed proteins, 547 proteins, were shared between ASD and control brains; of these 544 showed identical directionality of the differential expression between the brain regions for both ASD and controls (Fig. 2; Supplementary Table 3). These data indicate insignificant differences comparing BA19 vs. CB regional differentiation in ASD vs. control brains.

### Proteomic canonical pathway analysis reveals differentially enriched pathways in ASD vs. control brains, with BA19 and CB having both shared and distinctive pathway enrichments

To more fully understand the meanings of the differentially expressed proteins of each brain region, we used the Ingenuity Pathway Analysis® (IPA) bioinformatics tool to determine whether specific pathways and upstream regulators are enriched in ASD cases vs. controls. We found enrichment in ASD cases vs. controls for 58 and 86 canonical pathways for BA19 and CB, respectively, at p-value < 0.05, with 27 canonical pathways enriched in both BA19 and CB. Adjusting for multiple comparisons using Benjamini-Hochberg showed enrichment for 6 canonical pathways in BA19 and for 36 pathways in CB (Tables 1, 2 and Supplementary Table 4). Pathways enriched in both brain regions include glutamate receptor signaling and glutathione-mediated detoxification/oxidative stress. Pathways specifically enriched in one of the two regions include, for example, enrichment for Sertoli cell signaling in BA19 and enrichment for pathways of fatty acid, organic acid and branched chain amino acid metabolism in CB. Canonical pathway analysis using an independent bioinformatics tool, Reactome, highlighted major themes of the IPA-based analysis including glutamate (NMDA) receptor signaling in both brain regions and mitochondrial fatty acid and branched chain amino acid catabolism in CB (Supplementary Table 5).

**Table 1:** BA19-enriched canonical pathways from differentially expressed proteins (p < 0.05), adjusted for multiple comparisons.

Canonical Pathways	Enrichment Score (BH-adjusted)
FXR/RXR Activation	1.61
Glutamate Receptor Signaling	1.61
Clathrin-mediated Endocytosis Signaling	1.61
Heme Degradation	1.43
Sertoli Cell-Cell Junction Signaling	1.41
Glutathione-mediated Detoxification	1.41

Enrichment score of 1.3 equals a BH adjusted p-value of 0.05.

**Table 2:** CB-enriched canonical pathways from differentially expressed proteins (p < 0.05), adjusted for multiple comparisons.

Canonical Pathways	Enrichment Score (BH-adjusted)
Fatty Acid β-oxidation I	6.96
Glutaryl-CoA Degradation	6.14
Tryptophan Degradation III (Eukaryotic)	5.48
Isoleucine Degradation I	4.85
Valine Degradation I	4.43
Ketolysis	4.43
Aryl Hydrocarbon Receptor Signaling	2.93
Calcium Signaling	2.36
Xenobiotic Metabolism Signaling	2.36
Calcium Transport I	2.36
Phagosome Maturation	2.36
Ketogenesis	2.28
Amyotrophic Lateral Sclerosis Signaling	2.17
NRF2-mediated Oxidative Stress Response	2.17
Ethanol Degradation II	2.15
Mevalonate Pathway I	2.15
Noradrenaline and Adrenaline Degradation	2.03
14-3-3-mediated Signaling	1.96
Superpathway of Geranylgeranyldiphosphate Biosynthesis I (via Mevalonate)	1.94
LXR/RXR Activation	1.94
Parkinson’s Signaling	1.88
LPS/IL-1 Mediated Inhibition of RXR Function	1.79
Melatonin Signaling	1.78
Pyrimidine Deoxyribonucleotides *De Novo* Biosynthesis I	1.68
Tryptophan Degradation X (Mammalian, via Tryptamine)	1.68
Glutathione-mediated Detoxification	1.68
nNOS Signaling in Neurons	1.65
Ethanol Degradation IV	1.65
Serotonin Degradation	1.64
Synaptic Long Term Potentiation	1.6
PI3K Signaling in B Lymphocytes	1.54
PPARα/RXRα Activation	1.5
Calcium-induced T Lymphocyte Apoptosis	1.5
Superpathway of Cholesterol Biosynthesis	1.41
Glutamate Receptor Signaling	1.35
CREB Signaling in Neurons	1.3

Enrichment score of 1.3 equals a BH adjusted p-value of 0.05.

### Protein interaction network analyses differ between BA19 and CB

**Figure 3:**
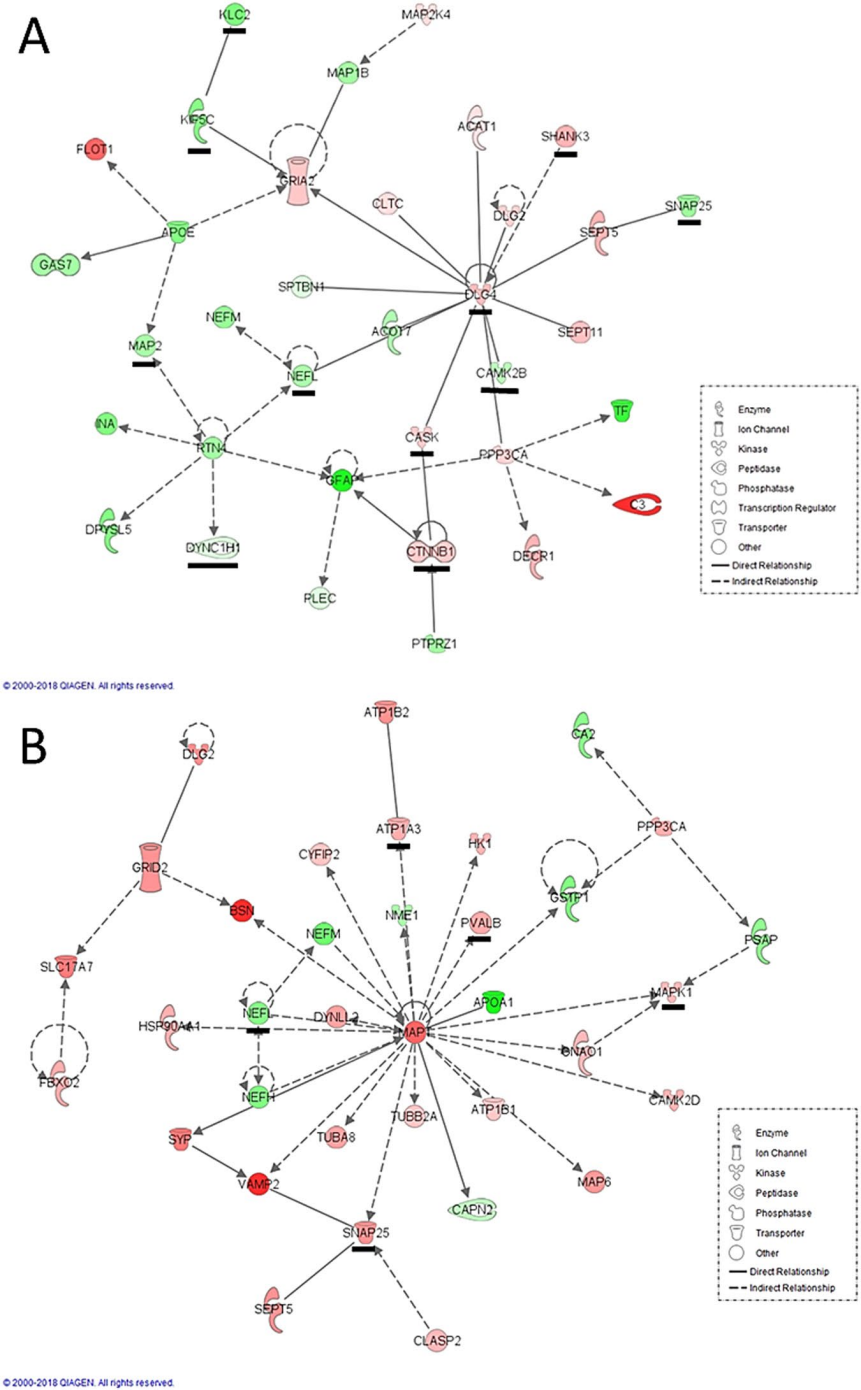
(**A**) BA19 empirical protein-protein interaction network generated from differentially expressed proteins. (**B**) CB empirical protein-protein interaction network generated from differentially expressed proteins. Green molecules are down-regulated in ASD and red up-regulated compared to controls. Underlined proteins have reported associations with ASD in SFARI Gene database (https://gene.sfari.org/).

We also sought to determine any protein-protein interaction networks that might also be differentially expressed between ASD cases and controls. Two significant interaction networks as determined by IPA, one for each brain region, are noted in Fig. 3. The BA19 network highlights proteins of synaptic function, cytoskeletal infrastructure of axon/neuron morphology, intracellular vesicle/organelle transport, and glutamatergic neurotransmission, with DLG4 as the major hub protein. The CB network highlights cytoskeletal and intracellular vesicular transport-related proteins, especially tubulins and tubulin-associated proteins, with MAPT as the major hub protein.

Networks in both brain regions share common features including: (1) up-regulation of multiple pre- and post-synaptic membrane or scaffolding proteins (eg, FLOT1, DLG2, DLG4, SHANK3 in BA19 and SYP, VAMP2, BSN, DLG2 in CB); (2) up-regulation of glutamatergic ion channels and related proteins (in BA19: FLOT1, CASK, GRIA2, DLG4 and DLG2; in CB: GRID2, SLC17A7 and DLG2); (3) up-regulation of proteins involved in intracellular calcium signaling (eg, PPP3CA, CASK in BA19 and CAMK2D, PPP3CA in CB); and (4) down-regulation of multiple neurofilament proteins (eg, NEFL, NEFM, INA in BA19 and NEFL, NEFM, NEFH in CB). A notable protein interaction difference between the networks is the up-regulation in CB of proteins comprising microtubules or involved in microtubule assembly or stability (eg, TUBA8, TUBB2A, MAP6, CLASP2, MAPT).

### Upstream regulator analysis suggests shared molecular dysregulation between ASD and neurodegeneration-associated proteins

Upstream regulators are molecules that are predicted to regulate the expression of the differentially expressed proteins. Using IPA, we noted 134 and 114 upstream regulators in BA19 and in CB, respectively, at p-value < 0.05; 70 of these were in common for both brain regions (Table 3, Supplementary Table 6). Functional assessment of these regulators revealed many that are involved in intracellular vesicular biology, synaptic biology, transcription, translation, and various metabolic functions. The most statistically significant upstream regulators are identical for both brain regions and include proteins associated with CNS neurodegenerative processes: MAPT, APP, PSEN1 and HTT.

**Table 3:** Top 10 upstream regulators for BA19 and CB that drive differential protein expression between ASD cases and controls based on downstream target expression changes.

Protein ID	Name	p-value	Associated Functions
**Upstream Regulators for BA19**			
MAPT	Microtubule-associated protein tau	3.39E-15	Microtubule assembly and stability
APP	Amyloid-beta A4 protein	7.45E-13	Neurite interactions, neuron adhesion, axonogenesis
PSEN1	Presenilin-1	3.36E-12	γ-secretase complex subunit
HTT	Huntington	5.17E-12	Microtubule-mediated transport or vesicle function
HDAC4	Histone deacetylase 4	1.12E-11	Histone deacetylation
MKNK1	MAP kinase-interacting serine/threonine-protein kinase 1	1.2E-09	Translation regulation, environmental stress response
BDNF	Brain-derived neurotrophic factor	3.18E-09	Peripheral and central nervous system development, synaptic function
ATN1	Atrophin-1	2.14E-07	Transcriptional corepressor
PPP3CA	Serine/threonine-protein phosphatase 2B catalytic subunit alpha isoform	2.86E-07	Calcineurin activity
PPARGC1A	Peroxisome proliferator-activated receptor gamma coactivator 1-alpha	6.29E-07	Transcriptional coactivator for PPARG,and TSH, metabolic responses to varying nutritional availability
**Upstream Regulators for CB**			
MAPT	Microtubule-associated protein tau	6.54E-09	Microtubule assembly and stability
APP	Amyloid-beta A4 protein	8.85E-08	Neurite interactions, neuron adhesion, axonogenesis
RTN4	Reticulon-4	1.10E-07	Neurite growth regulatory factor
PSEN1	Presenilin-1	2.35E-07	γ-secretase complex subunit
HTT	Huntington	2.22E-06	Microtubule-mediated transport or vesicle function
MTOR	Mechanistic Target Of Rapamycin	5.72E-06	Cellular metabolism, growth and survival
ROR1	Receptor Tyrosine Kinase Like Orphan Receptor 1	8.68E-05	Wnt signaling
ROR2	Receptor Tyrosine Kinase Like Orphan Receptor 2	8.68E-05	Osteoblast differentiation, bone formation, Wnt signaling
AGRN	Agrin	2.59E-04	Maintenance of the neuromuscular junction, post-synaptic differentiation
LPIN1	Phosphatidate phosphatase	2.59E-04	Fatty acid metabolism

P-values are derived from one-tailed Fisher’s exact test.

### Selective reaction monitoring (SRM) validates the  global, label-free proteomic analysis

To independently validate the global, label-free proteomic findings, we performed a targeted quantitative MS analysis of select peptides that are proxies for the 11 proteins of biological interest that are represented by 2 peptides, comparing both the directionality (ie, increased or decreased in ASD vs. controls) of the peptide noted in the unbiased, label-free analysis compared to the targeted analysis and whether the proteins were significantly differentially expressed by both label-free and SRM-based determinations. Of the 10 peptides selected for SRM validation analysis in BA19 that represented 5 proteins, 9 peptides validated in terms of directionality of expression and 3 of the 5 proteins validated in terms of significant differential expression for both types of analyses (p < 0.05). In CB samples, 12 peptides represented 6 proteins of interest; all 12 peptides validated in terms of directionality of expression and 5 of the 6 proteins validated in terms of significant differential expression for both of the two types of analyses (p < 0.05) (Supplementary Table 7). These data support the validity of the quantitative analyses produced by the unbiased, label-free MS analysis.

## Discussion

Intense efforts have been mounted in recent years to understand the biological basis of autism. These investigations have implicated dysfunction of numerous biochemical pathways and biological networks in autism biology; several investigative approaches, including cytogenomic and exome analyses of affected individuals and transcriptomic and metabolic analyses of ASD tissues, have been especially influential in shaping our current understanding of molecular mechanisms in ASD pathogenesis. This study describes the first global proteomic analysis of autistic brain. Our aim was to evaluate which proteins are differentially expressed in two functionally and geographically distinct regions of autism vs. control brain and, using these data, determine pathways and protein-protein interaction networks that are relevant to autism biology.

Current data indicate that there are a large number of known causes of autism, nearly all of which are uncommon or rare^9,10^. It is interesting that in this set of idiopathic autism brain samples 20 proteins in BA19 (13.7%) and 30 proteins in CB (15.7%) are differentially expressed in the examined ASD brains and are reported to have associations with either syndromic or non-syndromic autism in the SFARI Gene database^11^. Within the protein-protein interaction networks, 11 proteins in the BA19 network and 6 proteins in the CB network are represented in the SFARI database including, for example, SHANK3 and CTNNB1 in BA19 and GRID2 and ATP1A3 in CB (Fig. 2).

Based on the differential protein expression data, there is enrichment for pathways in both BA19 and CB relating to glutamate signaling and glutathione-mediated detoxification/oxidative stress. The protein-protein interaction network analyses for both brain regions indicate the importance of synapse formation/function, glutamatergic signaling, cytoskeletal support of axon/neuron structure and of intracellular vesicle trafficking, and the transduction of intracellular calcium signals. Involvement of these pathways and processes has been implicated in ASD pathogenesis^17–21^. For example, there are substantial data relating to glutamate and its receptors in a wide variety of neurobiological and behavioral findings associated with ASD^32,33^. Similarly, there are abundant data indicating evidence of oxidative stress in non-CNS tissues and brain region specific glutathione redox imbalance in ASD^34–36^.

The canonical pathway and protein network analyses also revealed brain region specific processes. Pathways that appear differentially expressed in the two regions include Sertoli cell signaling in BA19 and pathways of fatty acid, organic acid and branched chain amino acid metabolism in CB. The association with Sertoli cell signaling raises two issues: whether this finding may relate to earlier work suggesting an association with ASD and inhibin^37^ and the larger issue of a possible relationship with maleness, a strong but incompletely understood risk factor for autism^38^. Associations between both rare monogenic and common idiopathic forms of autism and branched chain amino acid metabolism are well established, as are associations with specific disturbances of organic acid metabolism^19,39–41^. Taken together, the differential protein expression data from human brain tissue provide direct support for shared autism-related pathobiology in the different brain regions studied here, as well as region-specific associations with autism.

In terms of networks, while both brain regions highlight synapse formation/function and glutamatergic signaling, the BA19-specific network highlights the function of DLG4 (PSD-95), a postsynaptic density protein that forms a multimeric scaffolding for the clustering of receptors, ion channels and associated signaling proteins, mediating NMDA receptor function and modulating synaptic plasticity via AMPA receptors^42^. The CB-specific network, while similar to BA19 in emphasizing cytoskeletal proteins that support axonal guidance/structure and intracellular vesicle trafficking, features in distinction to BA19 the up-regulation of tubulin and proteins associated with tubulin assembly and stability and a cerebellum-specific glutamate receptor, GRID2.

Striking data were noted regarding the upstream regulators, molecules that are predicted to drive the differential protein expression observed between ASD cases and controls through regulation of downstream targets. Upstream regulator analysis for both BA19 and CB brain regions showed highly significant enrichment for proteins that are central to many neurodegenerative disorders: MAPT (microtubule associated protein tau), APP (amyloid precursor protein), PSEN1 (presenilin-1) and HTT (huntington) (Table 3). Each of these proteins has multiple functions and it is possible that their actions as upstream regulators occur through different mechanisms^43–46^. However, a more parsimonious explanation is that there are overlapping protein interaction networks or other shared properties of these proteins that when perturbed result in converging pathogenetically relevant pathways; there are extensive data in support of this concept for many neurodegenerative disorders^47–49^. In terms of the upstream regulator data and a pathogenetic theory of autism, we hypothesize that perturbation of normal function of these proteins would impact part of a shared interactome, resulting in the broad CNS phenotype of autism. Two pathogenetic mechanisms are supported by the proteomic data of BA19 and CB and by the literature on shared interactomes and converging pathways in neurodegenerative disorders: impacts on synapse biology and on mitochondrial function^48,49^. There also are abundant data in support of disturbances of synaptic function and of mitochondrial bioenergetics in the pathogenesis of autism^15–19^. The data presented here suggest that the action(s) of several proteins that heretofore have had a thematic commonality of neurodegeneration also drive abnormal synaptic biology and/or mitochondrial dysfunction that is central to autism neurobiology.

There are both important similarities and differences of our findings and those of other studies using post-mortem ASD brains. Major themes of prior transcriptomic analyses of post-mortem brains, analyzing different brain regions, have included differential regulation of synaptic formation/function, immune response, mitochondrial activity and microglial functions^29–31,50,51^. Dysregulation in pathways governing cell number, cortical patterning and differentiation in young autism cases and dysregulation of signaling and repair pathways was noted in adult autism cases in another study^52^. Like these investigators, our proteomic analysis indicates dysregulation of synaptic biology and mitochondrial bioenergetics and provides limited support for immune response differences; our data do not affirm other themes noted above.

In addition, this work provides insights into whether post-mortem autistic brains involve attenuation of regional protein expression differences. Some prior ASD vs. control brain transcriptomic analyses noted that regional brain differentiation between frontal cortex and temporal cortex appeared to be attenuated in autism^30,31^, although other transcriptomic analyses evaluating possible attenuation of regional differentiation between ASD and control cerebellar and occipital cortex did not corroborate that finding^53^. The current proteomic data of cerebellar and occipital cortex show similar levels of differential expression between these two regions (Fig. 2; Supplementary Table 3), suggesting that the previously reported attenuation of regional brain gene expression in ASD may be anatomically specific. Consequently, this issue warrants further investigation, especially in terms of evaluating additional brain regions and the incorporation of proteomic methods.

There are significant preanalytic and analytic challenges in carrying out a proteomic investigation using human post-mortem brain tissue^54^. Additional challenges are faced when one studies post-mortem ASD brains as the number of available brain samples are limited. In addition, analyses of  ASD cases are potentially confounded by etiological heterogeneity, medical co-morbidities, and various treatments. Issues regarding ASD/control brain sample selection and our use of inclusion/exclusion criteria were described previously, including the rationale for use of idiopathic ASD brain samples to minimize confounding of data that can occur though inclusion of samples with diverse identifiable etiologies^55^.

Yet another challenge in neuroproteomics concerns the cellular complexity of brain tissue^24^. Although care was taken to obtain matched areas of brain for each case and paired control, it is possible that there could be differences in cellular composition between paired samples, either because of differences due to the different biological process affecting the ASD cases relative to controls or because of artifact or chance difference. To our knowledge, there have been no attempts to address this issue in proteomic analyses of ASD brain. We addressed this by evaluating the expression of cell type-specific markers between cases and controls for each major CNS cell type. We noted no significant differences in a pairwise analysis, although modest differences were noted for several markers using a regression model. Overall, the data suggest that there are no major cell population differences between ASD vs. control brain samples (Supplementary Fig. 1, Supplementary Table 8).

## Conclusions

This first proteomic evaluation of ASD human brains provides direct evidence of dysregulation of numerous proteins in two brain regions, BA19 and posterior inferior cerebellum, compared to controls. Bioinformatic analyses of the differentially expressed proteins of each of these brain regions suggests dysregulation of multiple pathways and protein interaction networks, especially implicating glutamatergic signaling. Overall, the proteomic data provide support for shared dysregulated pathways and upstream regulators for two brain regions in human ASD brain, suggesting a common ASD pathophysiology that has distinctive regional expression.

## Methods

### Subjects and samples

Brain samples were procured from the Autism Tissue Program (ATP, www.atpportal.org) through the Harvard Brain Tissue Resource Center (www.brainbank.mclean.org) and the National Institute for Child Health and Human Development Brain and Tissue Bank (www.btbank.org). The samples were used in accordance with the principles of the Declaration of Helsinki. Tissues were procured from a total of 9 ASD and 9 control subjects. Brain samples from both cases and controls are from the left posterior inferior cerebellar hemispheric cortex (CB) and BA19 (occipital) cortex. The samples from autism cases were obtained from validated cases of ASD who met detailed inclusion/exclusion criteria; controls were matched for gender, age, brain region and post-mortem interval^55^ (Supplementary Table 1). All 9 ASD brains were evaluated for *FMR1* nucleotide repeat expansion mutation, by two chromosomal microarray analyses, and with RNA-Sequencing based transcriptomic analyses; 8 of the 9 ASD brains were subject to deep sequencing of 78 known ASD candidate genes^29,55–57^. The result of this detailed characterization of the ASD cases was that 8 of 9 ASD cases did not have an identified pathogenic chromosomal lesion or monogenic disorder that might account for ASD; one case (ANO9714) has an etiologically relevant DNA sequence variant, *SCN2A* c.4543C > T, p.R1515*. Eight of the 9 ASD cases, therefore, meet criteria for idiopathic autism. Tissue for proteomic analyses was available from 9 ASD and 9 control subjects for CB and from 7 ASD and 6 control subjects for BA19.

### Global label-free expression proteomic analysis

Samples were rinsed in 100 mM Tris pH 8.0, transferred to a Covaris cryobag and frozen in liquid nitrogen, pulverized, transferred to glass vials, and then lysed with 2% SDS and 0.1% protease inhibitor cocktail (Sigma-Aldrich, St. Louis, MO). Samples were then sonicated, followed by filter-aided sample preparation detergent clean-up^58^. 10 mcg protein of each sample were digested with LysC for 1 hour and trypsin overnight at 37 °C. Reverse phase LC-MS/MS was performed as described^59^ except that 600 ng of peptide digests were loaded on the HPLC column and the gradient of solvent B ranged from 2% to 40% over 210 min.

Raw LC-MS/MS data files for each sample were processed using Rosetta Elucidator (Rosetta Biosoftware, Seattle, WA) (Version 3.3.01 SP4 25). Automated differential quantification of peptides was performed as previously described^60,61^. Briefly, LC-MS/MS raw data were imported, and for each MS spectrum profile of each LC-MS/MS run, chromatographic peaks and monoisotopic masses were extracted and aligned. Chromatographic peaks were first aligned by retention time and monoisotopic mass. Peak lists with the monoisotopic mass and corresponding MS/MS data were then generated for each sample and searched using Mascot (Matrix Science, London, UK; version 2.4.1). Resultant peptide identifications were imported into Elucidator and monoisotopic masses annotated with peptide identifications. The false discovery rate for protein identifications was calculated to be 2%. The MS/MS peak lists were searched by Mascot using the human UniProt database. Search settings were as follows: trypsin enzyme specificity; mass accuracy window for precursor ion, 25 ppm; mass accuracy window for fragment ions, 0.8 Da; variable modifications including carbamidomethlylation of cysteines, one missed cleavage and oxidation of methionine.

### Statistical methods

MS data were obtained for each region; these files contained intensity values, with rows corresponding to peptides and columns corresponding to the sample. Missing values were imputed using a weighted k-nearest neighbors algorithm^62^. Next, data were log_2_-transformed to improve symmetry and normal distribution approximation. Data were visualized with principal component analysis. One outlier was noted (BA19_6994) and removed from subsequent analyses. These preprocessing steps were performed using InfernoRDN^63^. As the principal component analysis showed clear separation of samples based on brain region, data from each brain region were separately used for subsequent statistical and bioinformatic analyses.

To increase statistical power, we treated individual peptides as observations of a given protein. Data were imported into the R statistical programming environment for subsequent analyses. Using the lme4 package^64^, a linear mixed effects model was applied to each protein with the following form:

$$intensity={\beta }_{1}\cdot CONDITION+{\beta }_{2}\cdot PMI+{\beta }_{3}\cdot AGE+{\beta }_{4}\cdot PEPTIDE+{\rm{\varepsilon }}$$

where intensity refers to the log_2_-transformed intensity values for each peptide observed for that protein, condition a binomial categorical variable (ASD or Control), PMI and age are continuous variables, peptide is a multilevel random effects categorical variable with the number of levels dependent on the protein, and ε is independent normally-distributed residual error with mean 0 and standard deviation σ. PMI data were unavailable for 3 samples, so these were assumed to be missing at random and imputed using predictive mean modeling, to allow use of all available intensity data in our model. For determination of proteins that varied significantly with changing PMI, we excluded these samples when running the model. A likelihood ratio was constructed for each coefficient in the model to assess its contribution to the variance in the data, and p-values were obtained. An additional analysis comparing regional contributions to differential expression within region, a binomial categorical variable, was run on the ASD and control datasets. P-values were adjusted using the false discovery rate (FDR) method described by Benjamini and Hochberg (BH)^65^. Multiple proteins were identified as cell type-specific markers. To assess potential contributions of cellular heterogeneity, these cell type-specific markers were assessed for differential expression through both the aforementioned regression model and separately with paired t-tests comparing expression changes between the paired autism and control samples. The pairs were compared using averages of the peptide intensities to reflect the specific protein concentration in each sample.

### Bioinformatics analyses

Differentially expressed proteins identified by at least two unique peptides underwent bioinformatic analysis. Network, pathway, and upstream regulator analyses were performed for each brain region with Ingenuity Pathway Analysis® (IPA, www.qiagen.com/ingenuity); proteins with unadjusted p-value < 0.05 were used for this purpose. Enrichment scores and p-values for canonical pathway and upstream regulator analyses were determined by a one-tailed Fisher’s exact test using the Ingenuity Knowledge Base as a reference. The latter is a database that is curated by expert reviewers that includes sets of member molecules of canonical pathways and downstream targets of upstream regulators, as well as all known interactions, direct and indirect, between proteins (http://pages.ingenuity.com/rs/ingenuity/images/IPA_data_sheet.pdf). Enrichment scores of above 1.3 correspond to a p-value < 0.05. Canonical pathway p-values were adjusted for multiple comparisons using the Benjamini-Hochberg (BH) procedure. Networks of protein-protein interactions were curated based on data from CNS and immune cell lines and tissues. The IPA bioinformatics tool generated interaction networks of the differentially expressed proteins through an iterative algorithm focused on interconnectivity between network members based on the Ingenuity Knowledge Base.

Results from IPA pathway analyses were validated using the open-source bioinformatics tool Reactome (https://reactome.org). P-values for Reactome-generated pathways from over-representation analysis were adjusted for multiple comparisons using the Benjamini Hochberg procedure.

### Targeted MS analysis with selective reaction monitoring

Validation of the global proteomic data was done through selected reaction monitoring MS to quantify the levels of several differentially expressed proteins noted in the global, label-free proteomic analysis. The targeted MS analysis was performed as described previously^66^. One or two peptides from each of 11 proteins of biological interest was synthesized (Sigma-Aldrich, St. Louis, MO) and used as a labeled internal standard that was spiked into each brain homogenate sample and used to quantify its unlabeled peptide analogue and thereby compute the concentration of the protein containing that peptide. Analysis of the selected peptides between the targeted and untargeted analyses was done using one-tailed Wilcoxon signed-rank tests. A p-value of 0.1 was considered significant given the nature of using Wilcoxon signed-rank tests on a small number of samples. Metrics assessed included consistency in directionality of expression between the label-free global vs. targeted analyses as well as whether statistical significance of differential expression was maintained. The peptides that were quantified, their amino acid sequences, and the proteins that they belong to are indicated in Supplementary Table 7.

## Supplementary information

Supplementary Tables and Figures

## Data Availability

The MS proteomics data have been deposited to the ProteomeXchange Consortium via the PRIDE^67^ repository with the dataset identifier PXD012755 and 10.6019/PXD012755. The code used for analysis is available upon request to the corresponding author.
