# Supplementary material for: Proteomic Investigations of Autism Brain Identify Known and Novel Pathogenetic Processes
Source: Sci Rep. 2019 Sep 11;9:13118. doi: 10.1038/s41598-019-49533-y (PMC6739414; doi:10.1038/s41598-019-49533-y)

# Proteomic Investigations of Autism Brain Identify Known and Novel Pathogenetic Processes

Joseph R. Abraham[1], Nicholas Szoko[1], John Barnard[2], Robert A. Rubin[3], Daniela Schlatzer[4], Kathleen Lundberg[4], Xiaolin Li[4], Marvin R. Natowicz[1,5,*]

[1]Cleveland Clinic Lerner College of Medicine of Case Western Reserve University, Cleveland, OH, 44195

[2]Department of Quantitative Health Sciences, Lerner Research Institute, Cleveland Clinic, Cleveland, OH, 44195

[3]Department of Mathematics, Whittier College, Whittier, CA, 90602

[4]Center for Proteomics, Case Western Reserve University, Cleveland, OH, 44106

[5]Pathology and Laboratory Medicine, Genomic Medicine, Neurological and Pediatrics Institutes, Cleveland Clinic, Cleveland OH, 44195

**[*]Correspondence:**
Marvin Natowicz, MD, PhD
Pathology and Laboratory Medicine Institute
Cleveland Clinic, LL-3
9500 Euclid Avenue
Cleveland, OH 44195
natowim@ccf.org

**Supplementary Tables and Figures**

Supplementary Table 1. Individual subject characteristics. PMI, postmortem interval; COD, cause of death.

| Subject ID: | Subject ATP ID: | Diagnosis | Regions | Age (years) | PMI (hours) | COD | Ethnicity | Medications |
|---|---|---|---|---|---|---|---|---|
| **6399** | **AN03345** | Autism | Cerebellum, BA19 | 2 | 4 | Hypoxia | White | None reported |
| **7078** | **AN17678** | Autism | Cerebellum | 11 | | Other | White | None reported |
| **6677** | **AN11989** | Autism | Cerebellum, BA19 | 30 | 16 | Cardiac | White | None reported |
| **6337** | **AN09730** | Autism | Cerebellum, BA19 | 22 | 25 | Hypoxia | White | Aripiprazole, lamotrigine, fish oil, multivitamin, zonisamide |
| **6994** | **AN08166** | Autism | Cerebellum, BA19 | 28 | 43 | Other | Unknown | Ziprasidone, cambamazepine, fexofenadine |
| **5666** | **AN19511** | Autism | Cerebellum, BA19 | 8 | 22.2 | Cancer | White | None reported |
| **6401** | **AN06420** | Autism | Cerebellum, BA19 | 39 | 14 | Cardiac | White | None reported |
| **5569** | **AN08873** | Autism | Cerebellum, BA19 | 5 | 25.5 | Hypoxia | White | Fluoxetine |
| **7090** | **AN09714** | Autism | Cerebellum, BA19 | 60 | 26.5 | Cancer | Unknown | Omeprazole, docusate, diazepam, phenobarbital, carbamazepine, guaifenesin |
| **5334** | **AN15622** | Control | Cerebellum, BA19 | 30 | 15 | Hypoxia | White | None reported |
| **6200** | **AN10723** | Control | Cerebellum, BA19 | 60 | 24.2 | Unknown | Unknown | None reported |
| **7561** | **AN05475** | Control | Cerebellum, BA19 | 39 | | Cardiac | Unknown | None reported |
| **5718** | **AN10833** | Control | Cerebellum, BA19 | 22 | 21.5 | Unknown | Unknown | None reported |
| **4678** | **UMB4670** | Control | Cerebellum, BA19 | 4 | 17 | Cardiac | White | None reported |
| **1453** | **BTB1453** | Control | Cerebellum, BA19 | 1 | 19 | Unknown | Black | None reported |
| **3328** | **BTB3228** | Control | Cerebellum | 11 | 20 | Other | Unknown | None reported |
| **4543** | **UMB4543** | Control | Cerebellum, BA19 | 28 | 13 | Other | White | None reported |
| **1860** | **UMB1860** | Control | Cerebellum, BA19 | 8 | 5 | Cardiac | White | None reported |

Supplementary Table 2. List of all differentially expressed proteins by brain region with p-value, adjusted p-value, and % expression in ASD (A) vs controls (C).

BA19 Differentially Expressed Proteins

| Accession ID | Protein symbol | P-values | Adjusted p-value | % Expression (A vs C) |
|---|---|---|---|---|
| P46821 | MAP1B | 1.86E-09 | 2.54E-06 | 84.1 |
| P49327 | FAS | 9.91E-08 | 6.76E-05 | 88.6 |
| P02787 | TF | 5.95E-07 | 2.71E-04 | 67.8 |
| P14136 | GFAP | 5.36E-06 | 1.83E-03 | 55.2 |
| P02675 | FGB | 6.84E-06 | 1.87E-03 | 296.3 |
| O75955 | FLOT1 | 1.03E-05 | 2.34E-03 | 147.8 |
| Q92561 | PHYHIP | 3.78E-05 | 7.37E-03 | 128.0 |
| P02679 | FGG | 5.90E-05 | 1.01E-02 | 302.8 |
| Q99961 | SH3GL1 | 1.12E-04 | 1.39E-02 | 133.0 |
| P08107 | HSPA1A | 1.30E-04 | 1.48E-02 | 149.4 |
| Q9P1U1 | ACTR3B | 1.66E-04 | 1.67E-02 | 82.2 |
| O00571 | DDX3X | 1.71E-04 | 1.67E-02 | 121.4 |
| Q9NZN3 | EHD3 | 1.92E-04 | 1.75E-02 | 120.2 |
| P01024 | C3 | 3.20E-04 | 2.73E-02 | 173.5 |
| P35222 | CTNNB1 | 3.67E-04 | 2.78E-02 | 83.5 |
| P11766 | ADH5 | 3.70E-04 | 2.78E-02 | 114.9 |
| P78352 | DLG4 | 3.87E-04 | 2.78E-02 | 118.6 |
| Q93050 | ATP6V0A1 | 4.36E-04 | 2.97E-02 | 118.9 |
| Q14011 | CIRBP | 5.03E-04 | 3.27E-02 | 50.5 |
| Q9Y6K8 | AK5 | 5.52E-04 | 3.43E-02 | 80.3 |
| P21266 | GSTM3 | 6.39E-04 | 3.78E-02 | 69.1 |
| Q92823 | NRCAM | 6.64E-04 | 3.78E-02 | 85.3 |
| P13693 | TPT1 | 7.61E-04 | 4.11E-02 | 81.6 |
| P07197 | NEFM | 9.19E-04 | 4.60E-02 | 81.8 |
| Q59EK9 | RUNDC3A | 9.43E-04 | 4.60E-02 | 126.5 |
| Q16352 | INA | 1.04E-03 | 4.84E-02 | 78.7 |
| P02671 | FGA | 1.06E-03 | 4.84E-02 | 325.1 |
| P09497 | CLTB | 1.19E-03 | 5.14E-02 | 63.6 |
| O00533 | CHL1 | 1.21E-03 | 5.14E-02 | 121.1 |
| Q16698 | DECR1 | 1.34E-03 | 5.54E-02 | 121.1 |
| P12814 | ACTN1 | 1.49E-03 | 5.98E-02 | 111.0 |
| Q9HB71 | CACYBP | 1.64E-03 | 6.39E-02 | 117.9 |
| Q8IXJ6 | SIRT2 | 2.11E-03 | 7.34E-02 | 78.3 |
| Q02878 | RPL6 | 2.14E-03 | 7.34E-02 | 86.7 |
| Q14254 | FLOT2 | 2.15E-03 | 7.34E-02 | 141.3 |
| Q14204 | DYNC1H1 | 2.50E-03 | 8.32E-02 | 93.4 |

| | | | | |
|---|---|---|---|---|
| P12955 | PEPD | 2.70E-03 | 8.77E-02 | 83.4 |
| P53396 | ACLY | 2.93E-03 | 9.09E-02 | 121.0 |
| O14994 | SYN3 | 2.95E-03 | 9.09E-02 | 83.9 |
| P23471 | PTPRZ1 | 3.00E-03 | 9.09E-02 | 88.5 |
| P02768 | ALB | 3.26E-03 | 9.54E-02 | 84.5 |
| P11137 | MTAP2 | 3.28E-03 | 9.54E-02 | 85.4 |
| P43003 | SLC1A3 | 3.56E-03 | 9.92E-02 | 194.7 |
| O14936 | CASK | 4.09E-03 | 1.09E-01 | 113.1 |
| Q15700 | DLG2 | 4.17E-03 | 1.10E-01 | 111.8 |
| P38646 | HSPA9 | 4.69E-03 | 1.19E-01 | 162.4 |
| P0C0L4 | C4A | 5.17E-03 | 1.22E-01 | 85.2 |
| Q99963 | SH3GL3 | 5.20E-03 | 9.93E-02 | 114.8 |
| P46439 | GSTM5 | 5.38E-03 | 1.22E-01 | 116.6 |
| Q6YN16 | HSDL2 | 5.54E-03 | 1.22E-01 | 30.4 |
| P00387 | CYB5R3 | 5.55E-03 | 1.22E-01 | 80.5 |
| Q08209 | PPP3CA | 5.84E-03 | 1.27E-01 | 112.4 |
| P49773 | HINT1 | 6.48E-03 | 1.36E-01 | 81.5 |
| P43007 | SLC1A4 | 6.63E-03 | 1.36E-01 | 122.0 |
| O75396 | SEC22B | 6.68E-03 | 1.36E-01 | 133.5 |
| P31937 | HIBADH | 7.26E-03 | 1.36E-01 | 139.6 |
| P55786 | NPEPPS | 7.46E-03 | 1.36E-01 | 91.1 |
| P07196 | NEFL | 8.14E-03 | 1.44E-01 | 84.9 |
| O43865 | AHCYL1 | 8.59E-03 | 1.50E-01 | 112.8 |
| O94985 | CLSTN1 | 8.98E-03 | 1.55E-01 | 78.3 |
| P31943 | HNRNPH1 | 9.36E-03 | 1.57E-01 | 116.3 |
| P13611 | VCAN | 9.84E-03 | 1.60E-01 | 68.5 |
| Q9BPU6 | DPYSL5 | 1.00E-02 | 1.60E-01 | 78.9 |
| Q9NQC3 | RTN4 | 1.02E-02 | 1.60E-01 | 83.6 |
| Q9Y3F4 | STRAP | 1.07E-02 | 1.64E-01 | 74.5 |
| P23634 | ATP2B4 | 1.09E-02 | 1.65E-01 | 76.9 |
| Q6PCE3 | PGM2L1 | 1.10E-02 | 1.65E-01 | 113.5 |
| O43776 | SYNC | 1.12E-02 | 1.67E-01 | 89.9 |
| P01034 | CST3 | 1.16E-02 | 1.71E-01 | 66.2 |
| Q9UH03 | SEPT3 | 1.18E-02 | 1.71E-01 | 112.3 |
| P51452 | DUSP3 | 1.30E-02 | 1.82E-01 | 76.7 |
| Q14832 | GRM3 | 1.31E-02 | 1.83E-01 | 129.8 |
| Q99719 | SEPT5 | 1.34E-02 | 1.84E-01 | 121.2 |
| P42704 | LRPPRC | 1.39E-02 | 1.88E-01 | 90.7 |
| Q9BRX8 | FAM213A | 1.43E-02 | 1.91E-01 | 82.7 |
| O60763 | USO1 | 1.50E-02 | 1.97E-01 | 125.8 |
| O60282 | KIF5C | 1.53E-02 | 1.97E-01 | 79.6 |

| | | | | |
|---|---|---|---|---|
| P01871 | IGHM | 1.58E-02 | 2.00E-01 | 72.0 |
| P45974 | USP5 | 1.58E-02 | 2.00E-01 | 85.7 |
| P49591 | SARS | 1.62E-02 | 2.01E-01 | 82.7 |
| Q9Y2A7 | NCKAP1 | 1.78E-02 | 2.17E-01 | 112.1 |
| P30041 | PRDX6 | 1.83E-02 | 2.20E-01 | 116.5 |
| P07305 | H1F0 | 1.86E-02 | 2.20E-01 | 74.9 |
| P30519 | HMOX2 | 1.88E-02 | 2.20E-01 | 86.4 |
| Q9UMF0 | ICAM5 | 1.89E-02 | 2.20E-01 | 126.0 |
| Q9NYU2 | UGGT1 | 1.98E-02 | 2.21E-01 | 109.2 |
| P62701 | RPS4X | 1.99E-02 | 2.21E-01 | 112.4 |
| Q00610 | CLTC | 1.99E-02 | 2.21E-01 | 89.3 |
| P30048 | PRDX3 | 2.05E-02 | 2.26E-01 | 110.7 |
| O95299 | NDUFA10 | 2.13E-02 | 2.29E-01 | 110.6 |
| O00154 | ACOT7 | 2.30E-02 | 2.45E-01 | 86.0 |
| P42262 | GRIA2 | 2.32E-02 | 2.45E-01 | 115.3 |
| P31153 | MAT2A | 2.46E-02 | 2.51E-01 | 73.4 |
| Q13509 | TUBB3 | 2.47E-02 | 2.51E-01 | 86.5 |
| Q01082 | SPTBN1 | 2.48E-02 | 2.51E-01 | 94.6 |
| P27797 | CALR | 2.49E-02 | 2.51E-01 | 111.9 |
| P30085 | CMPK1 | 2.55E-02 | 2.54E-01 | 112.6 |
| Q9NSE4 | IARS2 | 2.55E-02 | 2.54E-01 | 109.6 |
| P60880 | SNAP25 | 2.57E-02 | 2.54E-01 | 85.3 |
| P68104 | EEF1A1 | 2.61E-02 | 2.54E-01 | 86.2 |
| Q9NVA2 | SEP11 | 2.64E-02 | 2.54E-01 | 94.1 |
| O15020 | SPTBN2 | 2.64E-02 | 2.54E-01 | 117.5 |
| P78417 | GSTO1 | 2.69E-02 | 2.54E-01 | 82.8 |
| P45985 | MAP2K4 | 2.69E-02 | 2.54E-01 | 108.7 |
| P00338 | LDHA | 2.77E-02 | 2.54E-01 | 112.4 |
| P02649 | APOE | 2.80E-02 | 2.57E-01 | 85.6 |
| O15075 | DCLK1 | 2.88E-02 | 2.57E-01 | 119.4 |
| P01023 | A2M | 2.91E-02 | 2.57E-01 | 123.3 |
| Q9Y2Q0 | ATP8A1 | 2.95E-02 | 2.57E-01 | 75.8 |
| Q7L099 | RUFY3 | 2.95E-02 | 2.57E-01 | 89.5 |
| P62424 | RPL7A | 2.96E-02 | 2.57E-01 | 92.5 |
| P31689 | DNAJA1 | 2.97E-02 | 2.57E-01 | 88.0 |
| Q9NUQ9 | FAM49B | 3.00E-02 | 2.57E-01 | 131.6 |
| Q9BSJ8 | ESYT1 | 3.00E-02 | 2.57E-01 | 78.7 |
| Q9Y617 | PSAT1 | 3.01E-02 | 2.63E-01 | 78.8 |
| P08133 | ANXA6 | 3.03E-02 | 2.63E-01 | 77.9 |
| Q9UNM6 | PSMD13 | 3.08E-02 | 2.63E-01 | 112.6 |
| P62081 | RPS7 | 3.19E-02 | 2.63E-01 | 116.3 |

| | | | | |
|---|---|---|---|---|
| P04181 | OAT | 3.22E-02 | 2.63E-01 | 82.5 |
| Q9H0B6 | KLC2 | 3.26E-02 | 2.63E-01 | 88.8 |
| Q9BYB0 | SHANK3 | 3.31E-02 | 2.63E-01 | 121.6 |
| Q9UPR5 | SLC8A2 | 3.32E-02 | 2.63E-01 | 81.6 |
| P30043 | BLVRB | 3.37E-02 | 2.63E-01 | 86.8 |
| P32969 | RPL9 | 3.41E-02 | 2.63E-01 | 118.3 |
| P09429 | HMGB1 | 3.42E-02 | 2.63E-01 | 89.5 |
| Q9BPX5 | ARPC5L | 3.42E-02 | 2.63E-01 | 106.6 |
| P37837 | TALDO1 | 3.45E-02 | 2.63E-01 | 88.6 |
| P24752 | ACAT1 | 3.48E-02 | 2.63E-01 | 79.6 |
| Q02790 | FKBP4 | 3.50E-02 | 2.63E-01 | 88.2 |
| P52209 | PGD | 3.50E-02 | 2.63E-01 | 121.3 |
| Q13554 | CAMK2B | 3.58E-02 | 2.67E-01 | 87.3 |
| P62244 | RPS15A | 3.61E-02 | 2.68E-01 | 83.3 |
| P13645 | KRT10 | 3.81E-02 | 2.81E-01 | 76.3 |
| P50914 | RPL14 | 3.90E-02 | 2.83E-01 | 88.6 |
| Q15149 | PLEC | 4.05E-02 | 2.87E-01 | 64.5 |
| Q7Z3D6 | DGLUCY | 4.06E-02 | 2.87E-01 | 93.9 |
| P19105 | MYL12A | 4.13E-02 | 2.89E-01 | 125.4 |
| P63092 | GNAS | 4.20E-02 | 2.92E-01 | 113.1 |
| P22695 | UQCRC2 | 4.23E-02 | 2.92E-01 | 83.9 |
| O60861 | GAS7 | 4.23E-02 | 2.92E-01 | 89.8 |
| P13637 | ATP1A3 | 4.29E-02 | 2.92E-01 | 88.7 |
| P14866 | HNRNPL | 4.33E-02 | 2.93E-01 | 107.9 |
| P07900 | HSP90AA1 | 4.61E-02 | 3.06E-01 | 168.1 |
| Q14318 | FKBP8 | 4.64E-02 | 3.06E-01 | 82.3 |
| O75569 | PRKRA | 4.64E-02 | 3.06E-01 | 110.0 |
| P49006 | MARCKSL1 | 4.85E-02 | 3.15E-01 | 71.2 |

CB Differentially Expressed Proteins

| Accession ID | Protein symbol | P-value | Adjusted p-value | % Expression (A vs C) |
|---|---|---|---|---|
| P02768 | ALBU | 7.03E-13 | 1.01E-09 | 67 |
| Q14643 | ITPR1 | 2.31E-12 | 1.65E-09 | 136 |
| P02042 | HBD | 4.64E-11 | 2.21E-08 | 57 |
| Q01814 | AT2B2 | 3.32E-09 | 1.19E-06 | 125 |
| Q9NYI0 | PSD3 | 1.35E-08 | 3.86E-06 | 156 |
| P02787 | TRFE | 7.82E-08 | 1.86E-05 | 64 |
| P07196 | NFL | 2.18E-07 | 4.46E-05 | 75 |
| P19367 | HXK1 | 6.88E-07 | 1.23E-04 | 117 |
| Q7Z3D6 | GLUCM | 1.21E-06 | 1.92E-04 | 59 |
| P12036 | NFH | 2.36E-06 | 3.38E-04 | 74 |
| P17600 | SYN1 | 6.30E-06 | 8.19E-04 | 137 |

| | | | | |
|---|---|---|---|---|
| P16152 | CBR1 | 7.63E-06 | 9.10E-04 | 80 |
| Q01484 | ANK2 | 1.17E-05 | 1.24E-03 | 116 |
| P07197 | NFM | 1.21E-05 | 1.24E-03 | 71 |
| O15020 | SPTN2 | 1.32E-05 | 1.26E-03 | 116 |
| Q9H4G0 | E41L1 | 1.89E-05 | 1.69E-03 | 133 |
| Q02790 | FKBP4 | 2.48E-05 | 2.03E-03 | 127 |
| Q13554 | KCC2B | 2.56E-05 | 2.03E-03 | 124 |
| P05091 | ALDH2 | 3.17E-05 | 2.39E-03 | 74 |
| P06396 | GELS | 3.86E-05 | 2.76E-03 | 84 |
| Q9Y2J2 | E41L3 | 4.56E-05 | 3.11E-03 | 117 |
| P69905 | HBA | 5.09E-05 | 3.31E-03 | 62 |
| Q14204 | DYHC1 | 5.39E-05 | 3.33E-03 | 109 |
| Q99719 | SEPT5 | 5.58E-05 | 3.33E-03 | 125 |
| Q14168 | MPP2 | 2.26E-04 | 1.29E-02 | 124 |
| Q15149 | PLEC | 2.37E-04 | 1.30E-02 | 87 |
| P04040 | CATA | 2.76E-04 | 1.46E-02 | 68 |
| P07900 | HS90A | 3.23E-04 | 1.65E-02 | 118 |
| Q9P2U7 | VGLU1 | 4.47E-04 | 2.18E-02 | 131 |
| P00918 | CAH2 | 4.56E-04 | 2.18E-02 | 76 |
| Q9NSC5 | HOME3 | 4.77E-04 | 2.20E-02 | 130 |
| Q9Y6R1 | S4A4 | 4.95E-04 | 2.21E-02 | 125 |
| Q9UPA5 | BSN | 9.40E-04 | 3.96E-02 | 171 |
| P04264 | K2C1 | 1.01E-03 | 4.14E-02 | 133 |
| P10636 | TAU | 1.07E-03 | 4.24E-02 | 138 |
| P35219 | CAH8 | 1.24E-03 | 4.81E-02 | 132 |
| Q8N9I0 | SYT2 | 1.32E-03 | 4.95E-02 | 127 |
| Q9Y3E1 | HDGR3 | 1.35E-03 | 4.95E-02 | 124 |
| Q9ULP0 | NDRG4 | 1.45E-03 | 5.18E-02 | 127 |
| O75746 | CMC1 | 1.65E-03 | 5.77E-02 | 114 |
| Q08209 | PP2BA | 1.81E-03 | 5.97E-02 | 118 |
| P35908 | K22E | 1.82E-03 | 5.97E-02 | 139 |
| Q9P2R7 | SUCB1 | 1.84E-03 | 5.97E-02 | 114 |
| P07954 | FUMH | 1.92E-03 | 6.10E-02 | 88 |
| P07339 | CATD | 2.18E-03 | 6.79E-02 | 76 |
| P16615 | AT2A2 | 2.29E-03 | 6.97E-02 | 114 |
| P28482 | MK01 | 2.43E-03 | 7.23E-02 | 115 |
| P48664 | EAA4 | 2.52E-03 | 7.33E-02 | 151 |
| P37837 | TALDO | 2.56E-03 | 7.33E-02 | 117 |
| Q96FC7 | PHIPL | 2.77E-03 | 7.48E-02 | 126 |
| P02730 | B3AT | 2.81E-03 | 7.48E-02 | 61 |
| P63027 | VAMP2 | 2.82E-03 | 7.48E-02 | 173 |

| | | | | |
|---|---|---|---|---|
| O14531 | DPYL4 | 2.84E-03 | 7.48E-02 | 128 |
| Q15700 | DLG2 | 2.88E-03 | 7.48E-02 | 126 |
| Q13557 | KCC2D | 2.97E-03 | 7.48E-02 | 117 |
| Q9Y2A7 | NCKP1 | 2.98E-03 | 7.48E-02 | 115 |
| P30048 | PRDX3 | 3.52E-03 | 8.69E-02 | 79 |
| P38646 | GRP75 | 3.78E-03 | 9.15E-02 | 87 |
| Q08722 | CD47 | 3.97E-03 | 9.41E-02 | 121 |
| P24752 | THIL | 4.01E-03 | 9.41E-02 | 79 |
| Q13885 | TBB2A | 4.21E-03 | 9.49E-02 | 113 |
| P21926 | CD9 | 4.24E-03 | 9.49E-02 | 68 |
| Q92598 | HS105 | 4.60E-03 | 1.01E-01 | 128 |
| P00915 | CAH1 | 4.69E-03 | 1.01E-01 | 70 |
| P08237 | PFKAM | 4.74E-03 | 1.01E-01 | 114 |
| Q86VP6 | CAND1 | 5.40E-03 | 1.14E-01 | 109 |
| P13637 | AT1A3 | 5.64E-03 | 1.16E-01 | 119 |
| Q9UM19 | HPCL4 | 5.69E-03 | 1.16E-01 | 119 |
| Q9UKA9 | PTBP2 | 5.93E-03 | 1.18E-01 | 118 |
| Q969P0 | IGSF8 | 6.01E-03 | 1.18E-01 | 120 |
| P15531 | NDKA | 6.25E-03 | 1.21E-01 | 82 |
| P14415 | AT1B2 | 6.37E-03 | 1.21E-01 | 125 |
| Q02252 | MMSA | 6.53E-03 | 1.23E-01 | 87 |
| Q7Z6L0 | PRRT2 | 6.81E-03 | 1.25E-01 | 148 |
| P05026 | AT1B1 | 6.89E-03 | 1.25E-01 | 112 |
| Q06830 | PRDX1 | 6.96E-03 | 1.25E-01 | 86 |
| P00352 | AL1A1 | 7.54E-03 | 1.31E-01 | 123 |
| P21283 | VATC1 | 7.59E-03 | 1.31E-01 | 113 |
| P02545 | LMNA | 7.95E-03 | 1.33E-01 | 80 |
| P01857 | IGHG1 | 7.99E-03 | 1.33E-01 | 75 |
| P30043 | BLVRB | 8.56E-03 | 1.40E-01 | 54 |
| P52306 | GDS1 | 8.64E-03 | 1.40E-01 | 113 |
| P23634 | AT2B4 | 8.88E-03 | 1.42E-01 | 116 |
| P98179 | RBM3 | 9.02E-03 | 1.42E-01 | 67 |
| Q99714 | HCD2 | 9.07E-03 | 1.42E-01 | 88 |
| P40939 | ECHA | 9.13E-03 | 1.42E-01 | 87 |
| Q9BWM7 | SFXN3 | 9.39E-03 | 1.44E-01 | 118 |
| P22748 | CAH4 | 9.46E-03 | 1.44E-01 | 139 |
| Q9HB71 | CYBP | 9.59E-03 | 1.44E-01 | 113 |
| P51674 | GPM6A | 1.04E-02 | 1.49E-01 | 143 |
| P60880 | SNP25 | 1.05E-02 | 1.49E-01 | 125 |
| Q92823 | NRCAM | 1.05E-02 | 1.49E-01 | 111 |
| Q9NZ45 | CISD1 | 1.07E-02 | 1.49E-01 | 114 |

| | | | | |
|---|---|---|---|---|
| Q9UI12 | VATH | 1.08E-02 | 1.49E-01 | 116 |
| P17844 | DDX5 | 1.08E-02 | 1.49E-01 | 83 |
| P17677 | NEUM | 1.09E-02 | 1.49E-01 | 127 |
| P08247 | SYPH | 1.09E-02 | 1.49E-01 | 132 |
| P00568 | KAD1 | 1.10E-02 | 1.49E-01 | 76 |
| P02675 | FIBB | 1.16E-02 | 1.54E-01 | 155 |
| Q9UPR5 | NAC2 | 1.20E-02 | 1.57E-01 | 121 |
| Q9UH03 | SEPT3 | 1.21E-02 | 1.57E-01 | 117 |
| Q14576 | ELAV3 | 1.28E-02 | 1.63E-01 | 124 |
| Q8IV01 | SYT12 | 1.37E-02 | 1.72E-01 | 112 |
| P37235 | HPCL1 | 1.47E-02 | 1.82E-01 | 120 |
| P08670 | VIME | 1.50E-02 | 1.83E-01 | 60 |
| P09211 | GSTP1 | 1.52E-02 | 1.85E-01 | 74 |
| Q9NS86 | LANC2 | 1.54E-02 | 1.85E-01 | 135 |
| P49327 | FAS | 1.58E-02 | 1.88E-01 | 108 |
| P14136 | GFAP | 1.60E-02 | 1.88E-01 | 75 |
| P55084 | ECHB | 1.65E-02 | 1.89E-01 | 88 |
| O43424 | GRID2 | 1.66E-02 | 1.89E-01 | 125 |
| P68871 | HBB | 1.67E-02 | 1.89E-01 | 56 |
| Q9HDB5 | NRX3B | 1.68E-02 | 1.89E-01 | 120 |
| Q9UQ03 | COR2B | 1.70E-02 | 1.90E-01 | 119 |
| P08865 | RSSA | 1.72E-02 | 1.90E-01 | 88 |
| Q92561 | PHYIP | 1.74E-02 | 1.90E-01 | 120 |
| O14745 | NHRF1 | 1.75E-02 | 1.90E-01 | 81 |
| Q86UW7 | CAPS2 | 1.75E-02 | 1.90E-01 | 132 |
| P07099 | HYEP | 1.78E-02 | 1.92E-01 | 68 |
| P02765 | FETUA | 1.80E-02 | 1.93E-01 | 54 |
| Q9C005 | DPY30 | 1.87E-02 | 1.98E-01 | 70 |
| P08133 | ANXA6 | 1.88E-02 | 1.98E-01 | 88 |
| Q7Z3B1 | NEGR1 | 1.91E-02 | 1.98E-01 | 120 |
| P22392 | NDKB | 1.91E-02 | 1.98E-01 | 84 |
| P02647 | APOA1 | 1.92E-02 | 1.98E-01 | 49 |
| Q8TB36 | GDAP1 | 1.99E-02 | 2.04E-01 | 120 |
| O75122 | CLAP2 | 2.01E-02 | 2.04E-01 | 116 |
| P35527 | K1C9 | 2.10E-02 | 2.10E-01 | 126 |
| P22061 | PIMT | 2.11E-02 | 2.10E-01 | 76 |
| O43426 | SYNJ1 | 2.11E-02 | 2.10E-01 | 121 |
| Q9H2X9 | S12A5 | 2.24E-02 | 2.17E-01 | 119 |
| P43304 | GPDM | 2.25E-02 | 2.17E-01 | 112 |
| Q9NY65 | TBA8 | 2.25E-02 | 2.17E-01 | 121 |
| P55809 | SCOT1 | 2.26E-02 | 2.17E-01 | 108 |

| | | | | |
|---|---|---|---|---|
| P17655 | CAN2 | 2.28E-02 | 2.17E-01 | 86 |
| O95865 | DDAH2 | 2.34E-02 | 2.21E-01 | 81 |
| Q92597 | NDRG1 | 2.36E-02 | 2.21E-01 | 77 |
| Q9P2K5 | MYEF2 | 2.40E-02 | 2.23E-01 | 116 |
| P20472 | PRVA | 2.44E-02 | 2.23E-01 | 119 |
| P28161 | GSTM2 | 2.50E-02 | 2.27E-01 | 88 |
| Q9ULU8 | CAPS1 | 2.57E-02 | 2.31E-01 | 119 |
| Q13367 | AP3B2 | 2.67E-02 | 2.37E-01 | 119 |
| Q96E17 | RAB3C | 2.67E-02 | 2.37E-01 | 133 |
| O60268 | K0513 | 2.69E-02 | 2.38E-01 | 150 |
| Q96ID5 | IGS21 | 2.73E-02 | 2.40E-01 | 133 |
| P07237 | PDIA1 | 2.82E-02 | 2.45E-01 | 88 |
| P42126 | ECI1 | 2.87E-02 | 2.47E-01 | 79 |
| P05023 | AT1A1 | 2.98E-02 | 2.54E-01 | 108 |
| P00738 | HPT | 3.05E-02 | 2.56E-01 | 181 |
| Q9UBQ7 | GRHPR | 3.05E-02 | 2.56E-01 | 88 |
| P14550 | AK1A1 | 3.15E-02 | 2.61E-01 | 93 |
| O14594 | NCAN | 3.18E-02 | 2.61E-01 | 152 |
| Q9NZN3 | EHD3 | 3.18E-02 | 2.61E-01 | 120 |
| Q14318 | FKBP8 | 3.20E-02 | 2.61E-01 | 136 |
| P31937 | 3HIDH | 3.23E-02 | 2.61E-01 | 83 |
| Q9UBB6 | NCDN | 3.32E-02 | 2.64E-01 | 110 |
| P07602 | SAP | 3.32E-02 | 2.64E-01 | 77 |
| Q9UKU0 | ACSL6 | 3.36E-02 | 2.66E-01 | 120 |
| Q92522 | H1X | 3.42E-02 | 2.69E-01 | 114 |
| P52757 | CHIO | 3.48E-02 | 2.71E-01 | 137 |
| A6NE02 | BTBDH | 3.50E-02 | 2.71E-01 | 144 |
| O95831 | AIFM1 | 3.53E-02 | 2.72E-01 | 85 |
| P09960 | LKHA4 | 3.57E-02 | 2.73E-01 | 114 |
| O14979 | HNRDL | 3.60E-02 | 2.74E-01 | 85 |
| Q8TCU6 | PREX1 | 3.71E-02 | 2.79E-01 | 126 |
| P05937 | CALB1 | 3.76E-02 | 2.82E-01 | 120 |
| P13591 | NCAM1 | 3.81E-02 | 2.82E-01 | 108 |
| Q96JE9 | MAP6 | 3.83E-02 | 2.82E-01 | 124 |
| Q9H6S3 | ES8L2 | 3.84E-02 | 2.82E-01 | 124 |
| P23471 | PTPRZ | 3.86E-02 | 2.82E-01 | 113 |
| Q09666 | AHNK | 3.87E-02 | 2.82E-01 | 74 |
| Q05193 | DYN1 | 3.96E-02 | 2.87E-01 | 114 |
| Q96FJ2 | DYL2 | 3.97E-02 | 2.87E-01 | 121 |
| Q99497 | PARK7 | 4.01E-02 | 2.89E-01 | 84 |
| Q9UK22 | FBX2 | 4.09E-02 | 2.91E-01 | 119 |
| P21266 | GSTM3 | 4.20E-02 | 2.96E-01 | 78 |

| | | | | |
|---|---|---|---|---|
| P30101 | PDIA3 | 4.23E-02 | 2.97E-01 | 89 |
| Q92777 | SYN2 | 4.32E-02 | 3.00E-01 | 116 |
| P09471 | GNAO | 4.34E-02 | 3.00E-01 | 116 |
| Q16836 | HCDH | 4.39E-02 | 3.00E-01 | 89 |
| P30084 | ECHM | 4.40E-02 | 3.00E-01 | 86 |
| P48735 | IDHP | 4.41E-02 | 3.00E-01 | 86 |
| P42765 | THIM | 4.45E-02 | 3.02E-01 | 76 |
| Q14194 | DPYL1 | 4.50E-02 | 3.03E-01 | 110 |
| Q9H2U2 | IPYR2 | 4.65E-02 | 3.08E-01 | 81 |
| Q92752 | TENR | 4.67E-02 | 3.08E-01 | 117 |
| O75363 | BCAS1 | 4.68E-02 | 3.08E-01 | 65 |
| Q96F07 | CYFP2 | 4.78E-02 | 3.14E-01 | 113 |
| P02679 | FIBG | 4.84E-02 | 3.16E-01 | 149 |
| P16401 | HIST1H1B | 0.048936 | 3.18E-01 | 67 |
| O00567 | NOP56 | 0.049988 | 3.20E-01 | 117 |

Supplementary Table 3. List of proteins differentially expressed regionally in both ASD and Control datasets. Bolded proteins represent the 3 of 547 proteins that did not show directionality concordance in regional expression.

| UniProt Protein | Log Expr ASD | Log Expr Controls | P-value ASD | P-value Controls | UniProt Protein | Log Expr ASD | Log Expr Controls | P-value ASD | P-value Controls |
|---|---|---|---|---|---|---|---|---|---|
| O00154 | -0.71 | -1.02 | 5.85E-11 | 2.76E-11 | P50395 | -0.40 | -0.51 | 7.86E-10 | 6.93E-18 |
| O00429 | -1.28 | -1.44 | 1.55E-24 | 1.82E-27 | P50897 | 0.45 | 0.83 | 9.41E-03 | 8.06E-06 |
| O00483 | -0.88 | -0.54 | 7.41E-11 | 2.63E-04 | P50991 | -0.13 | -0.21 | 4.04E-02 | 1.26E-03 |
| O00499 | -0.79 | -1.14 | 4.43E-13 | 9.71E-16 | P50993 | -0.65 | -0.68 | 6.01E-22 | 3.53E-27 |
| O00764 | -0.87 | -1.02 | 4.29E-07 | 1.54E-12 | P51148 | -0.68 | -0.66 | 4.18E-12 | 3.95E-12 |
| O14531 | -1.87 | -2.33 | 1.78E-18 | 3.48E-16 | P51649 | -0.44 | -0.47 | 1.29E-04 | 1.12E-05 |
| O14561 | -0.51 | -0.32 | 1.69E-03 | 9.57E-04 | P51970 | -0.35 | -0.27 | 1.42E-03 | 2.09E-02 |
| O14594 | -1.71 | -2.50 | 5.08E-14 | 5.62E-08 | P51991 | 2.03 | 2.20 | 1.89E-19 | 1.86E-22 |
| O14745 | 0.75 | 0.98 | 8.09E-05 | 4.73E-06 | P52209 | -0.41 | -0.54 | 5.30E-05 | 2.40E-09 |
| O14818 | -0.34 | -0.32 | 7.55E-03 | 6.04E-06 | P52272 | 1.58 | 1.62 | 1.47E-25 | 6.44E-24 |
| O14979 | 0.96 | 1.14 | 5.85E-10 | 1.13E-14 | P52306 | -0.35 | -0.62 | 7.56E-07 | 2.10E-14 |
| O14980 | 0.82 | 0.58 | 5.47E-10 | 1.88E-09 | P52565 | -1.10 | -0.65 | 1.38E-06 | 3.44E-04 |
| O15020 | 0.93 | 0.79 | 1.60E-12 | 8.94E-11 | P53396 | 0.23 | 0.18 | 8.51E-03 | 3.71E-02 |
| O15144 | -0.32 | -0.24 | 3.89E-04 | 8.92E-03 | P53680 | -1.09 | -1.15 | 1.73E-08 | 1.33E-10 |
| O15540 | 1.97 | 1.99 | 1.55E-06 | 3.31E-07 | P54289 | -1.40 | -1.44 | 3.23E-16 | 3.19E-15 |
| O43236 | 0.99 | 0.51 | 5.06E-08 | 9.96E-03 | P54578 | -1.26 | -1.15 | 4.21E-10 | 7.04E-10 |
| O43301 | -1.69 | -1.74 | 2.16E-40 | 1.92E-54 | P55084 | 0.45 | 0.63 | 3.11E-05 | 2.92E-05 |
| O43390 | 1.80 | 1.68 | 6.29E-26 | 1.98E-20 | P55087 | -1.04 | -1.01 | 4.46E-03 | 1.70E-03 |
| O43426 | -0.94 | -1.34 | 2.87E-08 | 6.03E-12 | P55209 | -0.97 | -0.85 | 4.01E-10 | 1.04E-08 |
| O43491 | -0.82 | -0.79 | 2.59E-09 | 2.18E-04 | P55786 | -0.32 | -0.47 | 4.87E-08 | 8.88E-15 |
| O43676 | -0.89 | -0.71 | 8.29E-05 | 3.31E-03 | P55795 | 2.04 | 2.28 | 6.39E-10 | 5.20E-11 |
| O43678 | -0.86 | -0.54 | 1.91E-03 | 9.73E-04 | P55809 | -0.85 | -0.78 | 3.52E-23 | 3.83E-18 |
| O43681 | -0.45 | -0.49 | 8.72E-06 | 7.98E-06 | P56134 | -0.37 | -0.27 | 2.42E-05 | 1.62E-02 |
| O43707 | -0.70 | -0.43 | 2.94E-06 | 1.27E-02 | P59998 | -0.58 | -0.72 | 5.44E-06 | 8.04E-05 |
| O43776 | -0.44 | -0.48 | 5.85E-03 | 1.20E-05 | P60033 | 1.13 | 0.98 | 1.63E-06 | 2.52E-04 |
| O43813 | -0.37 | -0.62 | 1.32E-03 | 5.63E-09 | P60174 | 0.60 | 0.36 | 3.88E-21 | 1.92E-06 |
| O43837 | -0.45 | -0.40 | 2.90E-05 | 3.42E-06 | P60201 | -0.61 | -1.51 | 2.78E-03 | 8.42E-13 |
| O43865 | 0.49 | 0.76 | 1.00E-05 | 4.10E-11 | P60660 | 0.71 | 0.85 | 8.29E-06 | 1.39E-04 |
| O43920 | -0.78 | -0.56 | 1.24E-04 | 2.01E-04 | P60709 | -0.76 | -0.62 | 6.33E-11 | 5.91E-10 |
| O60262 | -1.01 | -1.46 | 4.00E-05 | 6.30E-05 | P60880 | -0.71 | -1.36 | 1.87E-11 | 4.62E-29 |
| O60313 | -0.71 | -0.61 | 4.74E-08 | 4.16E-05 | P60953 | -0.67 | -0.70 | 4.19E-06 | 3.67E-09 |
| O60506 | 0.50 | 0.67 | 1.05E-05 | 1.09E-07 | P60981 | -0.30 | -0.31 | 1.40E-02 | 8.24E-04 |
| O60641 | -0.95 | -0.88 | 1.75E-14 | 1.90E-10 | P61019 | -0.72 | -0.74 | 9.77E-07 | 3.00E-06 |
| O60814 | 2.93 | 3.07 | 6.66E-39 | 1.73E-45 | P61088 | -0.53 | -0.62 | 2.28E-03 | 2.32E-04 |
| O60888 | 0.67 | 0.82 | 3.64E-02 | 1.35E-02 | P61106 | -0.51 | -0.45 | 8.06E-06 | 1.73E-08 |
| O75061 | -1.15 | -1.32 | 1.62E-14 | 1.53E-15 | P61158 | -0.52 | -0.66 | 2.67E-02 | 2.45E-04 |
| O75083 | -0.75 | -0.63 | 1.03E-18 | 2.45E-15 | P61163 | -1.30 | -1.41 | 5.79E-07 | 8.50E-06 |

| | | | | | | | | | |
|---|---|---|---|---|---|---|---|---|---|
| O75122 | -0.71 | -0.91 | 2.06E-05 | 7.12E-08 | P61201 | -0.80 | -0.85 | 3.41E-08 | 3.28E-07 |
| O75251 | -0.69 | -0.55 | 8.67E-06 | 1.06E-03 | P61204 | -0.61 | -0.73 | 2.94E-06 | 3.78E-08 |
| O75306 | -1.00 | -0.48 | 8.77E-09 | 4.60E-04 | P61421 | -0.84 | -0.89 | 1.05E-09 | 2.60E-10 |
| O75323 | -0.53 | -0.35 | 1.11E-03 | 3.70E-03 | P61586 | -0.83 | -0.89 | 4.89E-04 | 2.15E-08 |
| O75367 | 3.25 | 3.32 | 5.98E-29 | 2.19E-28 | P61764 | -0.66 | -0.76 | 7.35E-17 | 7.18E-25 |
| O75390 | -0.38 | -0.33 | 2.61E-05 | 3.92E-05 | P61978 | 1.18 | 1.31 | 2.53E-15 | 5.13E-19 |
| O75475 | 2.77 | 2.59 | 1.41E-12 | 7.39E-13 | P61981 | -0.93 | -1.01 | 4.70E-26 | 2.94E-27 |
| O75489 | -1.12 | -0.76 | 3.73E-09 | 1.00E-09 | P62140 | -0.75 | -0.77 | 1.34E-10 | 1.16E-06 |
| O75506 | 0.43 | 0.48 | 8.14E-03 | 6.13E-03 | P62158 | -1.16 | -0.78 | 1.95E-11 | 3.07E-08 |
| O75508 | -1.39 | -2.33 | 1.90E-04 | 2.41E-06 | P62258 | -0.37 | -0.43 | 2.11E-07 | 1.83E-09 |
| O75746 | -0.41 | -0.55 | 3.00E-10 | 8.66E-15 | P62714 | -0.60 | -0.59 | 6.66E-05 | 3.54E-07 |
| O75781 | -2.15 | -1.88 | 2.04E-05 | 3.56E-06 | P62745 | -1.28 | -1.52 | 4.88E-12 | 4.23E-15 |
| O75947 | 0.69 | 0.72 | 8.21E-07 | 8.09E-07 | P62760 | -1.15 | -1.19 | 6.35E-13 | 9.91E-17 |
| O94760 | 0.36 | 0.15 | 8.32E-06 | 2.32E-02 | P62805 | 2.73 | 2.70 | 1.89E-24 | 1.60E-36 |
| O94811 | -2.19 | -2.46 | 7.98E-34 | 3.14E-27 | P62820 | -0.41 | -0.44 | 6.75E-04 | 2.57E-04 |
| O94819 | -0.92 | -1.39 | 1.84E-05 | 1.17E-05 | P62834 | -0.49 | -0.63 | 4.90E-05 | 3.86E-06 |
| O94826 | -1.20 | -1.20 | 1.17E-12 | 4.54E-12 | P62873 | -0.35 | -0.54 | 1.39E-07 | 9.60E-14 |
| O94856 | -0.65 | -1.10 | 1.14E-11 | 4.09E-18 | P62879 | -0.54 | -0.48 | 2.42E-04 | 8.23E-06 |
| O94925 | -1.21 | -1.10 | 1.12E-25 | 3.41E-24 | P63000 | -0.88 | -1.31 | 1.42E-03 | 9.66E-08 |
| O94973 | -0.94 | -0.81 | 4.72E-39 | 3.39E-37 | P63010 | -1.20 | -1.00 | 2.91E-27 | 6.35E-32 |
| O95292 | -0.89 | -1.23 | 2.61E-06 | 9.74E-12 | P63027 | -0.81 | -1.42 | 1.53E-02 | 3.04E-06 |
| O95299 | -0.72 | -0.32 | 8.20E-11 | 6.81E-06 | P63096 | -0.77 | -1.04 | 2.60E-05 | 3.72E-10 |
| O95319 | -0.67 | -0.48 | 3.31E-03 | 3.00E-02 | P63098 | -0.74 | -0.82 | 8.52E-09 | 4.38E-12 |
| O95670 | -0.68 | -1.23 | 9.77E-04 | 6.90E-04 | P63104 | -1.04 | -0.93 | 6.03E-12 | 4.42E-12 |
| O95674 | 0.58 | 0.56 | 1.59E-05 | 2.13E-07 | P63215 | -1.40 | -1.58 | 2.64E-04 | 3.07E-05 |
| O95782 | -1.41 | -1.37 | 2.95E-40 | 2.14E-37 | P67936 | 0.52 | 0.61 | 3.53E-02 | 2.00E-02 |
| O96000 | -1.01 | -0.72 | 2.83E-07 | 6.46E-06 | P68366 | -1.38 | -1.63 | 1.15E-27 | 1.64E-30 |
| P00338 | -0.35 | -0.44 | 1.92E-11 | 5.11E-11 | P68400 | -0.45 | -0.24 | 9.59E-03 | 2.24E-03 |
| P00352 | 3.04 | 2.55 | 1.70E-49 | 3.56E-32 | P68402 | 0.30 | 0.37 | 1.73E-02 | 2.95E-05 |
| P00367 | -1.23 | -1.08 | 1.33E-37 | 6.97E-34 | P68431 | 2.60 | 2.69 | 1.01E-29 | 3.31E-26 |
| P00395 | -0.62 | -0.54 | 2.60E-06 | 2.85E-04 | **P68871** | -0.34 | 0.64 | 1.79E-02 | 4.66E-04 |
| P00492 | -0.92 | -1.01 | 3.37E-24 | 7.52E-29 | **P69905** | -0.44 | 0.41 | 4.31E-04 | 3.29E-03 |
| P00505 | -0.51 | -0.28 | 2.78E-07 | 1.52E-05 | P78324 | -1.55 | -1.67 | 1.14E-23 | 2.20E-17 |
| P00558 | -0.10 | -0.14 | 3.34E-02 | 2.46E-05 | P78352 | -1.60 | -1.52 | 1.85E-11 | 2.01E-11 |
| P00918 | -1.13 | -0.55 | 2.72E-11 | 1.64E-02 | P78357 | -1.46 | -1.68 | 8.80E-13 | 2.70E-13 |
| P01834 | 0.92 | 1.43 | 7.79E-06 | 2.33E-05 | P78371 | -0.14 | -0.18 | 3.92E-02 | 1.31E-03 |
| P01857 | 0.43 | 1.05 | 1.71E-02 | 9.00E-05 | P78417 | -0.31 | -0.39 | 6.62E-04 | 5.22E-05 |
| P02545 | 0.78 | 0.97 | 1.16E-05 | 3.55E-03 | P78559 | -1.35 | -1.44 | 1.29E-29 | 3.67E-47 |
| P02647 | 1.73 | 2.30 | 3.82E-04 | 9.93E-04 | P80723 | -1.87 | -2.14 | 2.18E-30 | 2.95E-33 |
| P02686 | -0.90 | -1.02 | 4.87E-03 | 1.50E-07 | P84085 | -0.33 | -0.38 | 6.05E-04 | 1.39E-04 |
| P02730 | 0.50 | 1.49 | 4.23E-02 | 1.11E-05 | P99999 | -0.39 | -0.37 | 4.36E-03 | 1.27E-02 |
| P02768 | 0.30 | 0.84 | 1.86E-03 | 1.41E-17 | Q00610 | -0.84 | -0.70 | 1.21E-83 | 4.10E-85 |
| P02787 | -0.59 | -0.33 | 8.25E-06 | 1.25E-02 | Q00765 | -1.72 | -1.58 | 2.09E-12 | 1.59E-13 |

| | | | | | | | | | |
|---|---|---|---|---|---|---|---|---|---|
| P03905 | -0.75 | -0.61 | 9.94E-04 | 1.26E-04 | Q00839 | 1.60 | 1.77 | 4.50E-20 | 3.72E-37 |
| P04075 | -0.88 | -0.93 | 3.04E-20 | 1.51E-28 | Q01082 | -0.63 | -0.66 | 5.73E-56 | 2.69E-73 |
| P04179 | -0.58 | -0.32 | 7.01E-05 | 2.29E-02 | Q01105 | 0.80 | 1.24 | 2.84E-02 | 6.68E-06 |
| P04216 | -3.27 | -3.16 | 5.73E-20 | 5.96E-25 | Q01469 | 1.33 | 1.72 | 1.51E-07 | 9.30E-12 |
| P04264 | 1.07 | 0.56 | 1.27E-09 | 7.21E-03 | Q01484 | -0.37 | -0.65 | 1.54E-10 | 3.05E-23 |
| P04271 | 3.00 | 2.84 | 2.45E-16 | 7.02E-15 | Q01518 | -0.59 | -0.49 | 2.59E-13 | 1.31E-08 |
| P04350 | -0.58 | -0.93 | 2.56E-13 | 1.50E-27 | Q01813 | -0.83 | -1.16 | 3.29E-24 | 2.12E-34 |
| P04406 | -0.41 | -0.52 | 4.81E-08 | 3.02E-12 | Q01814 | -0.33 | -0.50 | 5.25E-10 | 3.69E-14 |
| P04632 | 0.77 | 0.53 | 5.40E-07 | 1.03E-02 | Q02218 | -0.59 | -0.43 | 3.09E-11 | 5.39E-05 |
| P04899 | -0.40 | -0.54 | 2.83E-04 | 6.51E-05 | Q02750 | -0.97 | -0.82 | 1.02E-04 | 1.83E-05 |
| P04908 | 2.90 | 2.84 | 3.21E-21 | 4.68E-21 | Q02790 | 0.91 | 0.58 | 1.87E-06 | 4.56E-04 |
| P05023 | -0.74 | -0.85 | 1.33E-20 | 3.18E-31 | Q02978 | -0.56 | -0.49 | 1.10E-06 | 1.78E-04 |
| P05026 | -1.13 | -1.29 | 1.11E-22 | 1.16E-23 | Q03252 | 2.31 | 2.35 | 1.02E-22 | 3.24E-23 |
| P05091 | -0.68 | -0.30 | 1.15E-11 | 5.53E-05 | Q04837 | 0.53 | 0.58 | 4.54E-02 | 6.87E-03 |
| P05141 | -0.52 | -0.47 | 7.02E-09 | 2.94E-09 | Q04917 | -0.72 | -0.65 | 1.53E-07 | 2.94E-05 |
| P05166 | 0.68 | 1.01 | 7.78E-10 | 1.12E-06 | Q05193 | -0.94 | -1.11 | 2.89E-26 | 7.89E-29 |
| P06748 | 1.74 | 2.01 | 2.16E-20 | 1.99E-21 | Q05329 | -1.26 | -1.37 | 1.49E-05 | 4.90E-04 |
| P07108 | 1.73 | 1.49 | 1.74E-17 | 1.89E-16 | Q05639 | -0.40 | -0.29 | 9.26E-07 | 1.34E-03 |
| P07195 | -0.22 | -0.32 | 3.03E-04 | 3.29E-06 | Q06124 | -0.74 | -0.90 | 5.94E-05 | 1.40E-08 |
| P07196 | -0.85 | -0.94 | 2.18E-18 | 2.44E-21 | Q07960 | -0.93 | -0.97 | 8.90E-06 | 1.97E-05 |
| P07197 | -0.98 | -1.02 | 4.29E-16 | 4.35E-21 | Q08209 | -1.23 | -1.40 | 6.09E-21 | 2.48E-24 |
| P07237 | 0.97 | 1.57 | 3.13E-06 | 3.05E-13 | Q08211 | 1.44 | 1.63 | 2.67E-23 | 5.19E-29 |
| P07305 | 3.20 | 3.23 | 5.79E-29 | 2.20E-28 | Q08722 | -0.74 | -1.11 | 3.06E-09 | 1.92E-09 |
| P07437 | -1.56 | -1.80 | 1.20E-36 | 3.26E-44 | Q10567 | -0.88 | -0.78 | 4.69E-42 | 1.56E-35 |
| P07737 | 0.76 | 0.76 | 2.80E-07 | 3.31E-08 | Q12860 | -0.80 | -1.01 | 3.46E-23 | 5.85E-33 |
| P07814 | -0.54 | -0.46 | 3.07E-05 | 1.14E-04 | Q12905 | 1.35 | 1.48 | 8.05E-18 | 1.02E-24 |
| P07900 | -0.17 | -0.47 | 2.54E-04 | 3.09E-26 | Q13200 | -1.11 | -0.90 | 3.49E-10 | 2.55E-12 |
| P07910 | 1.85 | 2.02 | 1.34E-21 | 4.73E-24 | Q13303 | -2.04 | -2.23 | 1.83E-12 | 8.44E-13 |
| P08107 | 0.28 | 0.56 | 7.42E-03 | 4.42E-09 | Q13367 | -0.53 | -0.71 | 1.91E-05 | 1.83E-04 |
| P08133 | -1.19 | -1.04 | 3.02E-25 | 8.12E-21 | Q13423 | -1.67 | -1.41 | 1.19E-34 | 3.45E-27 |
| P08195 | -0.85 | -0.70 | 7.10E-15 | 7.09E-16 | Q13509 | -1.52 | -1.59 | 6.93E-26 | 4.96E-32 |
| P08237 | -0.33 | -0.46 | 3.04E-04 | 8.57E-08 | Q13526 | -1.06 | -1.11 | 2.66E-03 | 4.12E-05 |
| P08238 | -0.29 | -0.49 | 1.05E-04 | 7.58E-14 | Q13555 | -1.66 | -2.01 | 1.50E-15 | 2.84E-20 |
| P08247 | -1.30 | -1.45 | 1.30E-09 | 9.26E-09 | Q13557 | 0.63 | 0.25 | 4.67E-05 | 4.52E-02 |
| P08559 | -0.99 | -0.98 | 1.51E-06 | 6.64E-09 | Q13642 | 0.42 | 0.29 | 5.76E-04 | 7.30E-03 |
| P08574 | -0.22 | -0.27 | 1.25E-02 | 1.69E-02 | Q13813 | -0.24 | -0.28 | 1.51E-09 | 9.89E-17 |
| P08758 | 0.40 | 0.81 | 3.84E-04 | 1.18E-06 | Q13825 | -0.75 | -0.41 | 2.31E-06 | 2.44E-02 |
| P08865 | 0.29 | 0.46 | 1.99E-03 | 2.17E-03 | Q13838 | 1.55 | 1.55 | 1.31E-13 | 8.01E-14 |
| P09104 | 0.45 | 0.19 | 1.56E-06 | 3.29E-02 | Q13885 | -1.12 | -1.31 | 8.09E-15 | 1.59E-14 |
| P09382 | 0.32 | 0.63 | 3.08E-02 | 4.50E-04 | Q14011 | 2.44 | 1.99 | 1.80E-07 | 8.05E-10 |
| P09417 | -0.65 | -1.17 | 1.24E-04 | 5.89E-10 | Q14019 | 0.40 | 0.51 | 4.93E-03 | 2.32E-03 |
| P09429 | 2.88 | 2.85 | 4.13E-15 | 1.58E-16 | Q14103 | 1.47 | 1.21 | 1.22E-05 | 1.64E-05 |
| P09471 | -0.85 | -1.03 | 6.47E-36 | 1.29E-33 | Q14141 | -0.71 | -0.66 | 1.54E-08 | 7.04E-04 |

| | | | | | | | | | |
|---|---|---|---|---|---|---|---|---|---|
| P09496 | -0.49 | -0.35 | 1.07E-03 | 1.29E-02 | Q14194 | -0.85 | -0.97 | 3.04E-21 | 2.27E-23 |
| P09497 | -0.90 | -0.62 | 3.18E-08 | 6.04E-04 | Q14195 | -0.78 | -0.82 | 4.91E-13 | 4.83E-09 |
| P09543 | -1.06 | -1.65 | 1.52E-16 | 1.15E-40 | Q14203 | -0.73 | -0.96 | 1.80E-03 | 1.63E-06 |
| P09622 | -0.61 | -0.37 | 5.48E-07 | 7.29E-05 | Q14204 | -0.15 | -0.37 | 4.34E-06 | 1.37E-31 |
| P09651 | 2.00 | 2.23 | 3.95E-19 | 9.65E-26 | Q14643 | 3.72 | 3.66 | 1.61E-36 | 1.62E-28 |
| P09669 | -1.30 | -0.82 | 7.17E-06 | 3.84E-04 | Q14847 | -1.30 | -1.22 | 4.59E-11 | 3.27E-11 |
| P09936 | -0.89 | -0.93 | 2.10E-15 | 2.13E-18 | Q14894 | -0.65 | -0.46 | 2.22E-06 | 1.06E-04 |
| P09972 | 1.33 | 1.03 | 1.49E-39 | 1.18E-30 | Q15019 | -1.77 | -1.69 | 3.28E-22 | 1.82E-13 |
| P0CG47 | 0.80 | 0.37 | 4.14E-06 | 4.24E-03 | Q15056 | 0.86 | 0.89 | 2.17E-03 | 4.05E-04 |
| P10412 | 3.44 | 3.31 | 4.65E-29 | 4.50E-30 | Q15102 | 1.95 | 2.17 | 5.84E-10 | 6.47E-11 |
| P10515 | -0.40 | -0.30 | 6.71E-03 | 4.65E-03 | Q15149 | -0.36 | -0.22 | 1.09E-08 | 6.08E-03 |
| P10636 | -1.57 | -2.10 | 1.33E-20 | 8.62E-26 | Q15181 | -1.10 | -1.35 | 3.68E-04 | 1.53E-04 |
| P10915 | -1.88 | -2.06 | 8.42E-09 | 2.72E-09 | Q15185 | 0.33 | 0.36 | 1.77E-02 | 1.04E-03 |
| P11021 | 0.27 | 0.47 | 1.09E-04 | 9.91E-07 | Q15257 | -0.50 | -0.41 | 5.73E-03 | 7.38E-06 |
| P11137 | -2.51 | -2.68 | 8.95E-48 | 2.23E-48 | Q15365 | 0.22 | 0.34 | 1.84E-05 | 2.93E-06 |
| P11142 | -0.34 | -0.46 | 1.11E-11 | 5.28E-20 | Q15366 | 0.28 | 0.45 | 2.68E-03 | 1.73E-10 |
| P11177 | -0.46 | -0.48 | 9.42E-07 | 3.33E-07 | Q15435 | -0.58 | -0.73 | 1.23E-13 | 3.59E-16 |
| P11216 | 0.61 | 0.45 | 1.33E-09 | 6.86E-08 | Q15555 | -1.19 | -1.40 | 1.22E-09 | 3.36E-10 |
| P11217 | -0.30 | -0.31 | 3.73E-02 | 3.88E-02 | Q15700 | -0.73 | -0.93 | 1.27E-10 | 1.13E-12 |
| P11233 | -0.80 | -1.07 | 5.89E-11 | 1.03E-14 | Q15717 | 3.01 | 2.97 | 1.10E-12 | 6.80E-15 |
| P11586 | -1.47 | -1.25 | 2.45E-09 | 4.17E-05 | Q16143 | -0.88 | -1.53 | 6.86E-04 | 3.84E-08 |
| P11766 | -0.37 | -0.34 | 4.25E-04 | 6.01E-04 | Q16181 | -0.91 | -0.93 | 3.21E-07 | 4.11E-09 |
| P11940 | 0.35 | 0.43 | 1.00E-02 | 2.13E-03 | Q16352 | -0.77 | -1.29 | 3.02E-12 | 1.23E-29 |
| P12111 | 0.88 | 1.29 | 1.54E-02 | 3.03E-02 | Q16531 | 0.39 | 0.22 | 2.92E-05 | 4.53E-02 |
| P12236 | 0.73 | 0.85 | 3.28E-03 | 2.63E-07 | Q16543 | -0.47 | -0.48 | 1.90E-03 | 6.23E-04 |
| P12532 | 0.45 | 0.42 | 4.96E-06 | 1.65E-05 | Q16555 | -0.47 | -0.81 | 3.42E-13 | 5.24E-31 |
| P12956 | 1.75 | 1.83 | 1.31E-21 | 2.30E-22 | Q16566 | 1.65 | 1.22 | 6.69E-06 | 2.49E-04 |
| P13010 | 1.82 | 1.91 | 2.71E-20 | 5.35E-23 | Q16623 | -3.22 | -2.82 | 7.89E-13 | 5.28E-10 |
| P13073 | -1.02 | -1.05 | 2.24E-09 | 1.86E-13 | Q16658 | -2.51 | -2.50 | 3.44E-36 | 5.94E-32 |
| P13489 | 0.32 | 0.39 | 8.35E-04 | 2.26E-06 | Q16774 | -0.27 | -0.27 | 7.70E-03 | 3.66E-02 |
| P13591 | -0.55 | -0.65 | 6.24E-14 | 9.21E-14 | Q16795 | -0.61 | -0.41 | 9.61E-07 | 1.55E-05 |
| **P13611** | 0.53 | -0.71 | 1.18E-03 | 9.23E-04 | Q16798 | -0.65 | -0.95 | 2.40E-04 | 1.75E-05 |
| P13637 | -0.56 | -0.92 | 3.04E-15 | 7.75E-20 | Q16799 | -0.92 | -1.03 | 3.39E-07 | 3.75E-15 |
| P13639 | -0.30 | -0.23 | 1.18E-07 | 2.85E-05 | Q16864 | -0.95 | -1.22 | 2.66E-05 | 6.67E-08 |
| P13693 | -0.77 | -0.83 | 2.83E-04 | 5.51E-08 | Q16891 | -0.49 | -0.36 | 1.72E-08 | 1.73E-05 |
| P13804 | 0.37 | 0.73 | 7.45E-03 | 6.04E-06 | Q53GQ0 | 0.38 | 0.38 | 4.44E-03 | 4.60E-02 |
| P14136 | 1.38 | 0.79 | 1.04E-12 | 5.27E-05 | Q5SSJ5 | 1.97 | 1.96 | 1.12E-13 | 7.48E-15 |
| P14314 | 0.56 | 0.82 | 1.79E-03 | 5.34E-04 | Q6P587 | -0.54 | -0.45 | 3.98E-08 | 2.08E-04 |
| P14415 | 0.74 | 0.58 | 1.44E-06 | 8.70E-03 | Q6UWR7 | -1.03 | -1.48 | 1.94E-05 | 4.15E-12 |
| P14618 | -0.31 | -0.41 | 1.54E-06 | 5.36E-14 | Q71U36 | -0.58 | -0.91 | 3.17E-15 | 1.27E-42 |
| P14625 | -0.43 | -0.29 | 9.44E-05 | 2.98E-02 | Q7L0J3 | -1.62 | -1.58 | 7.05E-13 | 4.60E-11 |
| P14866 | 1.49 | 1.79 | 9.41E-20 | 1.32E-27 | Q7Z3B1 | -1.37 | -1.78 | 5.99E-10 | 9.48E-08 |
| P15104 | -1.49 | -1.24 | 1.02E-20 | 3.36E-13 | Q7Z3D6 | 0.57 | 0.67 | 3.99E-02 | 1.54E-02 |

| | | | | | | | | | |
|---|---|---|---|---|---|---|---|---|---|
| P16298 | -1.44 | -1.53 | 9.75E-10 | 5.51E-08 | Q7Z4S6 | -0.76 | -0.59 | 2.57E-02 | 1.45E-02 |
| P16615 | -0.60 | -0.69 | 2.95E-14 | 1.90E-22 | Q7Z6L0 | -0.55 | -1.28 | 2.09E-03 | 4.07E-06 |
| P17174 | -1.02 | -1.15 | 8.40E-33 | 2.11E-34 | Q86Y82 | -0.53 | -0.58 | 2.23E-03 | 2.53E-04 |
| P17600 | -1.13 | -1.49 | 1.19E-50 | 6.37E-48 | Q8IXJ6 | -1.10 | -1.63 | 5.21E-10 | 3.55E-20 |
| P17655 | 0.34 | 0.58 | 6.67E-04 | 1.25E-05 | Q8N111 | -0.70 | -0.58 | 9.46E-07 | 1.79E-08 |
| P17677 | -1.41 | -1.91 | 2.34E-10 | 7.79E-11 | Q8N126 | 0.73 | 0.48 | 6.86E-07 | 9.10E-07 |
| P17858 | -0.55 | -0.47 | 4.87E-06 | 9.07E-05 | Q8N3J6 | -1.43 | -1.70 | 1.09E-08 | 3.89E-10 |
| P18669 | -0.26 | -0.28 | 4.80E-05 | 1.49E-06 | Q8N573 | -1.51 | -1.79 | 1.68E-11 | 1.35E-12 |
| P18859 | 0.94 | 0.96 | 1.33E-02 | 2.58E-03 | Q8NCW5 | -0.53 | -0.34 | 3.13E-09 | 2.36E-05 |
| P19086 | -0.68 | -0.82 | 1.56E-03 | 7.10E-03 | Q8WUM4 | -1.23 | -0.91 | 2.70E-03 | 8.59E-06 |
| P19338 | 0.62 | 0.98 | 3.84E-02 | 5.50E-05 | Q8WXF7 | -1.62 | -1.70 | 4.30E-21 | 1.44E-31 |
| P19367 | -0.53 | -0.72 | 9.41E-29 | 6.24E-40 | Q92499 | 0.73 | 0.77 | 5.53E-10 | 8.62E-11 |
| P20020 | -1.86 | -1.84 | 5.54E-38 | 1.98E-24 | Q92561 | -0.33 | -0.26 | 1.52E-04 | 4.37E-02 |
| P21281 | -1.07 | -1.16 | 6.69E-24 | 5.53E-30 | Q92597 | -1.69 | -1.44 | 5.98E-17 | 7.48E-08 |
| P21283 | -0.84 | -1.05 | 1.16E-09 | 1.44E-12 | Q92598 | -1.97 | -2.45 | 6.90E-25 | 2.37E-21 |
| P21579 | -1.19 | -1.07 | 1.59E-24 | 3.51E-33 | Q92777 | -0.91 | -0.99 | 6.43E-26 | 5.55E-19 |
| P21796 | -0.94 | -0.91 | 1.05E-15 | 1.01E-22 | Q92823 | -0.83 | -1.15 | 2.08E-32 | 4.31E-29 |
| P21926 | -1.10 | -0.86 | 7.18E-04 | 3.72E-03 | Q92841 | 1.83 | 1.65 | 5.40E-11 | 4.81E-10 |
| P21964 | 0.36 | 0.64 | 6.87E-03 | 2.39E-03 | Q92945 | 1.59 | 1.79 | 2.82E-04 | 9.93E-10 |
| P22061 | -0.77 | -0.52 | 1.51E-02 | 1.52E-08 | Q93050 | -1.07 | -1.00 | 9.53E-17 | 2.03E-16 |
| P22392 | -0.82 | -0.55 | 4.15E-10 | 8.63E-07 | Q96AB3 | -0.87 | -0.41 | 8.97E-14 | 9.83E-03 |
| P22626 | 1.76 | 1.69 | 7.38E-18 | 1.04E-25 | Q96BM9 | -1.38 | -1.30 | 1.28E-05 | 1.40E-11 |
| P22676 | 5.12 | 5.24 | 9.19E-11 | 3.72E-15 | Q96C19 | -0.62 | -0.52 | 4.55E-02 | 1.36E-02 |
| P23246 | 2.23 | 2.24 | 9.52E-16 | 1.43E-16 | Q96CW1 | -1.01 | -0.98 | 8.22E-24 | 3.47E-32 |
| P23381 | -0.82 | -0.66 | 3.26E-03 | 1.85E-03 | Q96E17 | -1.64 | -1.82 | 1.37E-10 | 5.12E-09 |
| P23526 | -1.04 | -1.17 | 1.36E-10 | 1.13E-08 | Q96F07 | -0.84 | -0.83 | 1.81E-10 | 1.28E-16 |
| P23528 | -0.27 | -0.47 | 3.37E-02 | 1.59E-04 | Q96FC7 | -0.39 | -0.93 | 2.21E-06 | 1.48E-08 |
| P23634 | -0.97 | -1.17 | 9.20E-14 | 4.18E-13 | Q96GD0 | -1.22 | -1.12 | 7.83E-04 | 3.42E-03 |
| P24534 | 0.28 | 0.31 | 1.39E-02 | 2.01E-03 | Q96GW7 | -1.26 | -1.57 | 2.09E-12 | 2.76E-11 |
| P24539 | -0.30 | -0.31 | 4.78E-03 | 2.19E-03 | Q96JE9 | -1.35 | -1.56 | 5.80E-11 | 2.94E-11 |
| P25398 | 0.61 | 0.61 | 2.05E-04 | 1.12E-03 | Q96KP4 | -0.54 | -0.59 | 6.44E-05 | 1.09E-04 |
| P25705 | -0.39 | -0.27 | 3.80E-14 | 2.01E-08 | Q96QK1 | -0.59 | -0.54 | 6.54E-11 | 1.61E-08 |
| P25789 | -0.47 | -0.28 | 5.16E-07 | 7.08E-04 | Q99497 | 0.34 | 0.61 | 1.10E-02 | 1.82E-07 |
| P26038 | 0.70 | 0.93 | 4.14E-02 | 1.63E-02 | Q99623 | -0.77 | -0.59 | 4.64E-15 | 1.72E-10 |
| P26232 | -1.45 | -1.53 | 3.74E-15 | 3.05E-14 | Q99714 | 0.45 | 0.73 | 7.41E-08 | 1.87E-13 |
| P26641 | 0.45 | 0.41 | 1.79E-02 | 2.53E-02 | Q99719 | -1.50 | -1.64 | 1.26E-30 | 5.69E-25 |
| P27338 | -0.90 | -0.80 | 1.79E-06 | 3.68E-03 | Q99747 | -0.46 | -0.78 | 4.73E-06 | 2.02E-06 |
| P27361 | -0.57 | -0.89 | 4.49E-03 | 7.88E-06 | Q99798 | -0.27 | -0.27 | 7.34E-06 | 5.36E-09 |
| P27449 | -0.86 | -0.66 | 3.44E-06 | 5.62E-05 | Q99961 | -1.49 | -1.31 | 1.84E-06 | 2.75E-04 |
| P27695 | 1.82 | 1.84 | 6.33E-22 | 9.80E-23 | Q99963 | -1.04 | -1.01 | 1.14E-09 | 5.56E-09 |
| P27797 | 0.79 | 1.09 | 7.97E-11 | 3.19E-13 | Q9BPU6 | -0.66 | -0.95 | 9.03E-05 | 4.44E-07 |
| P28331 | -0.57 | -0.24 | 1.30E-11 | 4.74E-05 | Q9BRX8 | -1.13 | -0.99 | 9.19E-11 | 2.86E-06 |
| P29401 | 0.70 | 0.55 | 1.24E-31 | 1.60E-21 | Q9BT78 | -0.22 | -0.23 | 2.77E-02 | 9.87E-04 |

| | | | | | | | | | |
|---|---|---|---|---|---|---|---|---|---|
| P29966 | 0.87 | 0.91 | 5.15E-07 | 4.31E-11 | Q9BWM7 | 0.94 | 0.60 | 6.68E-10 | 6.61E-05 |
| P30042 | -0.81 | -0.53 | 1.08E-09 | 1.58E-06 | Q9BY11 | -1.71 | -1.83 | 1.09E-20 | 1.89E-19 |
| P30044 | 0.45 | 0.51 | 2.40E-07 | 4.10E-08 | Q9C040 | -0.39 | -0.56 | 7.04E-05 | 4.51E-07 |
| P30084 | -0.95 | -0.37 | 3.83E-09 | 2.42E-02 | Q9H115 | -0.30 | -0.69 | 9.08E-04 | 2.74E-09 |
| P30101 | 0.48 | 0.81 | 4.01E-10 | 1.43E-14 | Q9H492 | -1.24 | -1.03 | 2.45E-07 | 6.49E-05 |
| P30153 | -0.55 | -0.67 | 5.88E-15 | 1.16E-20 | Q9H4G0 | -1.04 | -1.33 | 5.33E-12 | 5.86E-18 |
| P30531 | -1.51 | -1.59 | 3.33E-06 | 8.40E-06 | Q9H7Z7 | -0.47 | -0.29 | 1.39E-02 | 3.82E-02 |
| P31040 | -1.49 | -1.17 | 2.07E-12 | 3.58E-11 | Q9H8H3 | 0.69 | 0.95 | 2.24E-04 | 4.91E-03 |
| P31146 | -1.90 | -1.69 | 4.85E-16 | 4.64E-11 | Q9H936 | -1.05 | -1.02 | 3.28E-10 | 5.55E-08 |
| P31150 | -0.34 | -0.51 | 1.61E-05 | 7.14E-12 | Q9H9B4 | -0.53 | -0.52 | 1.46E-07 | 9.11E-08 |
| P31153 | 0.48 | 0.36 | 9.68E-05 | 1.42E-03 | Q9HCJ6 | 4.69 | 3.81 | 7.66E-10 | 1.12E-19 |
| P31689 | -0.74 | -0.84 | 1.22E-06 | 1.01E-07 | Q9HDC9 | 0.70 | 0.75 | 8.07E-07 | 1.91E-07 |
| P31930 | -0.34 | -0.22 | 4.71E-05 | 5.86E-03 | Q9NP72 | -0.33 | -0.45 | 8.09E-03 | 6.91E-04 |
| P31937 | 0.41 | 0.96 | 8.85E-04 | 1.18E-06 | Q9NP97 | 0.38 | 0.42 | 2.05E-02 | 4.25E-03 |
| P31943 | 1.30 | 1.47 | 5.86E-10 | 1.88E-09 | Q9NPJ3 | -1.30 | -1.14 | 1.18E-10 | 2.98E-06 |
| P31946 | -0.49 | -0.49 | 1.09E-03 | 1.08E-04 | Q9NQC3 | -1.06 | -1.21 | 1.28E-12 | 2.25E-17 |
| P31948 | 0.48 | 0.31 | 5.95E-06 | 2.40E-08 | Q9NQX3 | -1.13 | -0.66 | 1.44E-06 | 8.13E-04 |
| P32004 | 0.54 | 0.61 | 4.08E-09 | 5.94E-09 | Q9NS86 | -0.95 | -1.49 | 6.29E-04 | 7.46E-06 |
| P32119 | 0.32 | 0.57 | 2.96E-03 | 1.23E-07 | Q9NSD9 | -0.67 | -0.74 | 1.58E-10 | 1.46E-09 |
| P34932 | -0.75 | -0.79 | 5.16E-15 | 5.69E-17 | Q9NTK5 | -0.22 | -0.32 | 2.95E-02 | 3.10E-04 |
| P35222 | -0.39 | -0.22 | 6.57E-05 | 6.97E-03 | Q9NVA2 | -1.40 | -1.23 | 1.19E-28 | 5.29E-26 |
| P35232 | -0.94 | -0.43 | 1.11E-06 | 7.15E-04 | Q9NX63 | -0.37 | -0.26 | 2.31E-03 | 9.40E-03 |
| P35527 | 0.69 | 0.49 | 1.19E-03 | 4.31E-02 | Q9NYI0 | 1.38 | 0.81 | 9.06E-14 | 3.96E-05 |
| P35580 | -0.81 | -0.44 | 8.93E-09 | 1.29E-03 | Q9NZ45 | -0.82 | -0.91 | 1.41E-11 | 1.53E-14 |
| P35611 | -0.69 | -0.70 | 1.34E-10 | 2.50E-10 | Q9NZJ7 | -0.86 | -0.40 | 1.46E-03 | 4.61E-03 |
| P35612 | -1.13 | -1.13 | 4.61E-13 | 3.16E-18 | Q9NZN3 | -1.77 | -1.85 | 1.24E-17 | 3.00E-10 |
| P35613 | -0.82 | -0.75 | 7.34E-10 | 1.01E-05 | Q9NZR1 | -0.43 | -0.49 | 3.72E-06 | 1.33E-07 |
| P36542 | -0.42 | -0.31 | 1.21E-06 | 5.29E-06 | Q9P0L0 | -0.41 | -0.45 | 1.35E-04 | 1.20E-03 |
| P36543 | -0.94 | -0.92 | 7.18E-08 | 8.48E-12 | Q9P2R7 | -0.30 | -0.46 | 1.53E-04 | 6.48E-07 |
| P37837 | 1.83 | 1.20 | 1.06E-22 | 8.09E-17 | Q9P2U7 | -0.92 | -1.19 | 1.19E-11 | 1.11E-09 |
| P37840 | -0.66 | -0.96 | 8.32E-04 | 8.58E-06 | Q9UBB6 | -1.51 | -1.55 | 3.87E-48 | 6.30E-37 |
| P38117 | -0.63 | -0.49 | 6.09E-05 | 1.37E-04 | Q9UEY8 | 0.52 | 0.31 | 2.52E-06 | 1.17E-02 |
| P38606 | -0.76 | -0.89 | 1.50E-16 | 2.74E-23 | Q9UH03 | -1.06 | -1.17 | 2.90E-20 | 8.49E-17 |
| P39687 | 1.72 | 1.75 | 2.72E-07 | 4.05E-08 | Q9UI12 | -0.96 | -1.15 | 7.57E-18 | 8.15E-19 |
| P40123 | -2.41 | -2.24 | 3.66E-12 | 1.18E-13 | Q9UI15 | -0.93 | -1.07 | 5.79E-08 | 5.52E-13 |
| P40925 | -0.90 | -1.06 | 5.23E-19 | 1.09E-20 | Q9UKA9 | 1.46 | 1.19 | 7.99E-13 | 4.07E-09 |
| P40926 | -1.01 | -0.92 | 8.44E-49 | 1.73E-42 | Q9UKU0 | -0.54 | -0.73 | 8.52E-06 | 9.17E-05 |
| P40939 | 0.46 | 0.70 | 6.94E-08 | 4.37E-12 | Q9ULD0 | -0.48 | -0.42 | 8.29E-05 | 1.46E-03 |
| P41250 | -0.31 | -0.50 | 2.83E-05 | 9.99E-12 | Q9ULU8 | -0.74 | -0.84 | 7.74E-09 | 3.65E-05 |
| P42025 | -0.85 | -1.08 | 1.86E-11 | 3.40E-13 | Q9UM19 | 0.67 | 0.56 | 5.75E-06 | 1.72E-03 |
| P42658 | -1.01 | -0.93 | 1.94E-07 | 1.01E-02 | Q9UN36 | 1.18 | 1.01 | 6.19E-16 | 5.64E-12 |
| P43003 | 0.73 | 1.21 | 1.55E-03 | 7.86E-07 | Q9UN86 | -1.16 | -0.99 | 4.50E-09 | 2.86E-10 |
| P43004 | -3.49 | -3.46 | 1.83E-20 | 2.98E-15 | Q9UPA5 | -1.70 | -2.37 | 6.62E-09 | 2.52E-07 |

| | | | | | | | | | |
|---|---|---|---|---|---|---|---|---|---|
| P43034 | -0.31 | -0.40 | 6.40E-04 | 6.73E-05 | Q9UPN3 | 0.36 | 0.62 | 1.06E-02 | 2.89E-03 |
| P43121 | 2.85 | 2.61 | 1.69E-10 | 9.20E-10 | Q9UPR5 | 0.63 | 0.55 | 6.38E-08 | 2.21E-04 |
| P43243 | 1.81 | 1.80 | 2.37E-39 | 4.46E-37 | Q9UPY8 | -0.86 | -0.90 | 4.84E-07 | 1.77E-09 |
| P43304 | 0.56 | 0.41 | 2.49E-06 | 2.85E-03 | Q9UQ16 | 0.65 | 0.21 | 3.03E-11 | 3.99E-02 |
| P46459 | -0.96 | -1.07 | 3.27E-43 | 1.01E-53 | Q9UQ80 | 0.55 | 0.47 | 8.75E-07 | 5.77E-05 |
| P46821 | -0.42 | -0.76 | 5.96E-09 | 3.54E-25 | Q9UQB3 | -0.97 | -1.16 | 3.57E-06 | 4.61E-07 |
| P47755 | -0.43 | -0.42 | 3.72E-05 | 3.82E-08 | Q9UQM7 | -3.64 | -3.77 | 5.13E-36 | 4.21E-34 |
| P47756 | -0.49 | -0.58 | 9.23E-06 | 4.29E-08 | Q9Y224 | 1.25 | 1.45 | 2.52E-10 | 4.09E-10 |
| P48047 | -0.41 | -0.24 | 2.77E-06 | 1.38E-03 | Q9Y266 | -0.74 | -0.64 | 1.04E-05 | 9.02E-05 |
| P48147 | -1.50 | -1.50 | 2.34E-09 | 8.54E-09 | Q9Y277 | -0.51 | -0.51 | 1.13E-13 | 2.57E-19 |
| P48556 | -0.43 | -0.47 | 1.18E-03 | 4.36E-04 | Q9Y281 | -0.35 | -0.47 | 5.13E-03 | 1.23E-03 |
| P48643 | -0.36 | -0.35 | 2.10E-06 | 2.60E-06 | Q9Y285 | -0.97 | -1.14 | 2.12E-06 | 3.58E-08 |
| P48735 | -1.20 | -0.74 | 2.76E-36 | 4.28E-12 | Q9Y2A7 | -0.61 | -0.76 | 1.55E-04 | 1.17E-07 |
| P49189 | 0.41 | 0.43 | 1.98E-05 | 5.41E-06 | Q9Y2J2 | -0.70 | -1.02 | 6.69E-25 | 1.61E-31 |
| P49368 | -0.21 | -0.20 | 1.95E-03 | 1.85E-03 | Q9Y2J8 | -0.84 | -1.00 | 5.44E-07 | 1.72E-07 |
| P49411 | -0.25 | -0.17 | 2.27E-02 | 3.57E-02 | Q9Y4I1 | -0.93 | -1.04 | 2.91E-11 | 1.29E-12 |
| P49418 | -1.03 | -1.37 | 2.32E-10 | 3.23E-15 | Q9Y5K8 | -1.06 | -1.26 | 1.93E-10 | 4.69E-08 |
| P49419 | -0.75 | -0.73 | 1.31E-18 | 2.60E-12 | Q9Y617 | -0.31 | -0.63 | 1.25E-04 | 5.02E-06 |
| P49588 | -0.40 | -0.55 | 5.49E-07 | 2.54E-17 | Q9Y639 | -0.86 | -0.56 | 3.11E-05 | 1.58E-04 |
| P49758 | -1.67 | -1.65 | 6.47E-11 | 1.57E-07 | Q9Y6I3 | -1.15 | -0.98 | 7.57E-06 | 4.99E-08 |
| P49821 | -0.57 | -0.41 | 8.90E-11 | 1.80E-06 | Q9Y6R1 | 1.00 | 0.65 | 1.16E-05 | 1.71E-02 |
| P50213 | -0.52 | -0.44 | 2.89E-05 | 1.38E-05 | - | - | - | - | - |

Supplementary Table 4**.** Non-FDR adjusted enriched IPA-generated canonical pathways for BA19 and Cerebellum. An enrichment of 1.3 or greater represents a p-value < 0.05.

| **CANONICAL PATHWAYS - BA19** | **Enrichment** |
|---|---|
| FXR/RXR Activation | 3.81 |
| Glutamate Receptor Signaling | 3.72 |
| Clathrin-mediated Endocytosis Signaling | 3.59 |
| Heme Degradation | 3.28 |
| Sertoli Cell-Sertoli Cell Junction Signaling | 3.17 |
| Glutathione-mediated Detoxification | 3.09 |
| Phagosome Maturation | 2.9 |
| LXR/RXR Activation | 2.82 |
| Signaling by Rho Family GTPases | 2.76 |
| Acute Phase Response Signaling | 2.67 |
| Remodeling of Epithelial Adherens Junctions | 2.54 |
| Pentose Phosphate Pathway | 2.52 |
| EIF2 Signaling | 2.47 |
| Calcium Signaling | 2.44 |
| RhoA Signaling | 2.37 |
| tRNA Charging | 2.24 |
| Pyrimidine Ribonucleotides Interconversion | 2.24 |
| Glucocorticoid Receptor Signaling | 2.23 |
| Pyrimidine Ribonucleotides De Novo Biosynthesis | 2.18 |
| Huntington's Disease Signaling | 2.17 |
| nNOS Signaling in Neurons | 2.09 |
| Acetyl-CoA Biosynthesis III (from Citrate) | 2.03 |
| Pyrimidine Deoxyribonucleotides De Novo Biosynthesis I | 1.83 |
| Methionine Degradation I (to Homocysteine) | 1.83 |
| Protein Kinase A Signaling | 1.82 |
| Mitochondrial Dysfunction | 1.78 |
| Germ Cell-Sertoli Cell Junction Signaling | 1.78 |
| Amyotrophic Lateral Sclerosis Signaling | 1.75 |
| Cysteine Biosynthesis III (mammalia) | 1.75 |
| Palmitate Biosynthesis I (Animals) | 1.73 |
| Ascorbate Recycling (Cytosolic) | 1.73 |
| Fatty Acid Biosynthesis Initiation II | 1.73 |
| Arginine Degradation I (Arginase Pathway) | 1.73 |
| Formaldehyde Oxidation II (Glutathione-dependent) | 1.73 |
| Synaptic Long Term Potentiation | 1.67 |
| LPS/IL-1 Mediated Inhibition of RXR Function | 1.65 |
| Xenobiotic Metabolism Signaling | 1.64 |
| Gap Junction Signaling | 1.61 |
| NRF2-mediated Oxidative Stress Response | 1.59 |

| | |
|---|---|
| Complement System | 1.58 |
| Protein Ubiquitination Pathway | 1.57 |
| Pyruvate Fermentation to Lactate | 1.55 |
| S-adenosyl-L-methionine Biosynthesis | 1.55 |
| Role of NFAT in Cardiac Hypertrophy | 1.55 |
| Sirtuin Signaling Pathway | 1.48 |
| Aryl Hydrocarbon Receptor Signaling | 1.48 |
| Epithelial Adherens Junction Signaling | 1.47 |
| Arsenate Detoxification I (Glutaredoxin) | 1.43 |
| Proline Biosynthesis II (from Arginine) | 1.43 |
| Pentose Phosphate Pathway (Oxidative Branch) | 1.43 |
| Arginine Degradation VI (Arginase 2 Pathway) | 1.43 |
| Stearate Biosynthesis I (Animals) | 1.42 |
| Superpathway of Methionine Degradation | 1.42 |
| Salvage Pathways of Pyrimidine Ribonucleotides | 1.36 |
| Arginine Biosynthesis IV | 1.34 |
| Serine Biosynthesis | 1.34 |
| Pentose Phosphate Pathway (Non-oxidative Branch) | 1.34 |
| Citrulline Biosynthesis | 1.34 |
| **CANONICAL PATHWAYS - Cerebellum** | **Enrichment** |
| Fatty Acid β-oxidation I | 9.47 |
| Glutaryl-CoA Degradation | 8.35 |
| Tryptophan Degradation III (Eukaryotic) | 7.51 |
| Isoleucine Degradation I | 6.75 |
| Valine Degradation I | 6.19 |
| Ketolysis | 6.16 |
| Aryl Hydrocarbon Receptor Signaling | 4.59 |
| Calcium Signaling | 3.93 |
| Xenobiotic Metabolism Signaling | 3.9 |
| Calcium Transport I | 3.87 |
| Phagosome Maturation | 3.83 |
| Ketogenesis | 3.71 |
| Amyotrophic Lateral Sclerosis Signaling | 3.54 |
| NRF2-mediated Oxidative Stress Response | 3.53 |
| Ethanol Degradation II | 3.48 |
| Mevalonate Pathway I | 3.46 |
| Noradrenaline and Adrenaline Degradation | 3.31 |
| 14-3-3-mediated Signaling | 3.21 |
| Superpathway of Geranylgeranyldiphosphate Biosynthesis I (via Mevalonate) | 3.15 |
| LXR/RXR Activation | 3.15 |
| Parkinson's Signaling | 3.07 |
| LPS/IL-1 Mediated Inhibition of RXR Function | 2.96 |

| | |
|---|---|
| Melatonin Signaling | 2.93 |
| Pyrimidine Deoxyribonucleotides De Novo Biosynthesis I | 2.78 |
| Tryptophan Degradation X (Mammalian, via Tryptamine) | 2.78 |
| Glutathione-mediated Detoxification | 2.78 |
| nNOS Signaling in Neurons | 2.72 |
| Ethanol Degradation IV | 2.71 |
| Serotonin Degradation | 2.69 |
| Synaptic Long Term Potentiation | 2.63 |
| PI3K Signaling in B Lymphocytes | 2.56 |
| PPARα/RXRα Activation | 2.49 |
| Calcium-induced T Lymphocyte Apoptosis | 2.49 |
| Superpathway of Cholesterol Biosynthesis | 2.39 |
| Glutamate Receptor Signaling | 2.31 |
| CREB Signaling in Neurons | 2.25 |
| Role of NFAT in Cardiac Hypertrophy | 2.2 |
| Remodeling of Epithelial Adherens Junctions | 2.16 |
| Granzyme A Signaling | 2.13 |
| Neuropathic Pain Signaling In Dorsal Horn Neurons | 2.01 |
| Aldosterone Signaling in Epithelial Cells | 1.99 |
| GM-CSF Signaling | 1.98 |
| Pyrimidine Ribonucleotides Interconversion | 1.94 |
| Mitochondrial Dysfunction | 1.91 |
| Pyrimidine Ribonucleotides De Novo Biosynthesis | 1.88 |
| Histamine Degradation | 1.81 |
| B Cell Receptor Signaling | 1.81 |
| Lipid Antigen Presentation by CD1 | 1.76 |
| Oxidative Ethanol Degradation III | 1.76 |
| Salvage Pathways of Pyrimidine Ribonucleotides | 1.75 |
| Apoptosis Signaling | 1.73 |
| Amyloid Processing | 1.72 |
| Gap Junction Signaling | 1.72 |
| Synaptic Long Term Depression | 1.72 |
| FXR/RXR Activation | 1.7 |
| Glutathione Redox Reactions I | 1.67 |
| Putrescine Degradation III | 1.67 |
| Fatty Acid α-oxidation | 1.67 |
| Semaphorin Signaling in Neurons | 1.65 |
| Unfolded protein response | 1.63 |
| Huntington's Disease Signaling | 1.63 |
| TCA Cycle II (Eukaryotic) | 1.62 |
| β-alanine Degradation I | 1.62 |
| Phenylethylamine Degradation I | 1.62 |
| Palmitate Biosynthesis I (Animals) | 1.62 |

| | |
|---|---|
| Thyroid Hormone Biosynthesis | 1.62 |
| Fatty Acid Biosynthesis Initiation II | 1.62 |
| Fatty Acid β-oxidation III (Unsaturated, Odd Number) | 1.62 |
| GNRH Signaling | 1.61 |
| Thrombin Signaling | 1.61 |
| TR/RXR Activation | 1.57 |
| Acute Phase Response Signaling | 1.56 |
| Breast Cancer Regulation by Stathmin1 | 1.51 |
| Dopamine Degradation | 1.48 |
| Trehalose Degradation II (Trehalase) | 1.45 |
| D-glucuronate Degradation I | 1.45 |
| Glycerol-3-phosphate Shuttle | 1.45 |
| Lactose Degradation III | 1.45 |
| Nitric Oxide Signaling in the Cardiovascular System | 1.45 |
| iCOS-iCOSL Signaling in T Helper Cells | 1.42 |
| Chemokine Signaling | 1.38 |
| PKCθ Signaling in T Lymphocytes | 1.36 |
| Heme Degradation | 1.33 |
| Glutathione Redox Reactions II | 1.33 |
| Glioma Signaling | 1.32 |
| Role of Macrophages, Fibroblasts and Endothelial Cells in Rheumatoid Arthritis | 1.3 |

.

Supplementary Table 5. Reactome-generated canonical pathways for each brain region. P-values were generated from overrepresentation analysis.

| Reactome Pathway Analysis | | | | | |
|---|---|---|---|---|---|
| BA19 | | | Cerebellum | | |
| **Pathway name** | **p-value** | **FDR-adjusted p-value** | **Pathway name** | **p-value** | **FDR-adjusted p-value** |
| Unblocking of NMDA receptors, glutamate binding and activation | 1.10E-05 | 2.62E-03 | Ion homeostasis | 4.59E-09 | 3.00E-06 |
| Influenza Life Cycle | 1.23E-05 | 2.62E-03 | Erythrocytes take up oxygen and release carbon dioxide | 8.77E-09 | 3.00E-06 |
| HSP90 chaperone cycle for steroid hormone receptors (SHR) | 1.39E-05 | 2.62E-03 | Erythrocytes take up carbon dioxide and release oxygen | 9.42E-08 | 1.61E-05 |
| Post-translational protein phosphorylation | 1.66E-05 | 2.62E-03 | O2/CO2 exchange in erythrocytes | 9.42E-08 | 1.61E-05 |
| HSF1-dependent transactivation | 2.08E-05 | 2.62E-03 | Ion transport by P-type ATPases | 7.28E-07 | 9.90E-05 |
| Influenza Infection | 2.26E-05 | 2.62E-03 | Beta oxidation of hexanoyl-CoA to butanoyl-CoA | 2.24E-06 | 1.90E-04 |
| Long-term potentiation | 2.52E-05 | 2.62E-03 | Beta oxidation of lauroyl-CoA to decanoyl-CoA-CoA | 2.24E-06 | 1.90E-04 |
| Peptide chain elongation | 3.35E-05 | 3.05E-03 | Beta oxidation of octanoyl-CoA to hexanoyl-CoA | 2.24E-06 | 1.90E-04 |
| Attenuation phase | 4.30E-05 | 3.09E-03 | Beta oxidation of decanoyl-CoA to octanoyl-CoA-CoA | 4.58E-06 | 3.33E-04 |
| Selenocysteine synthesis | 4.54E-05 | 3.09E-03 | Neuronal System | 4.89E-06 | 3.33E-04 |
| Eukaryotic Translation Elongation | 4.89E-05 | 3.09E-03 | Neurexins and neuroligins | 1.33E-05 | 8.22E-04 |
| Regulation of Insulin-like Growth Factor (IGF) transport and uptake by Insulin-like Growth Factor Binding Proteins (IGFBPs) | 5.15E-05 | 3.09E-03 | Transmission across Chemical Synapses | 2.70E-05 | 1.54E-03 |
| Axon guidance | 6.09E-05 | 3.41E-03 | Cardiac conduction | 4.45E-05 | 2.31E-03 |
| Regulation of TLR by endogenous ligand | 1.38E-04 | 7.20E-03 | mitochondrial fatty acid beta-oxidation of saturated fatty acids | 4.83E-05 | 2.32E-03 |
| Infectious disease | 1.70E-04 | 8.14E-03 | Mitochondrial Fatty Acid Beta-Oxidation | 6.52E-05 | 2.56E-03 |
| Negative regulation of NMDA receptor-mediated neuronal transmission | 2.02E-04 | 9.11E-03 | L1CAM interactions | 6.56E-05 | 2.56E-03 |

| | | | | | |
|---|---|---|---|---|---|
| Selenoamino acid metabolism | 2.14E-04 | 9.21E-03 | Reduction of cytosolic Ca++ levels | 6.74E-05 | 2.56E-03 |
| Neutrophil degranulation | 2.64E-04 | 1.06E-02 | Caspase-mediated cleavage of cytoskeletal proteins | 6.74E-05 | 2.56E-03 |
| Eukaryotic Translation Termination | 3.06E-04 | 1.16E-02 | Muscle contraction | 9.69E-05 | 3.49E-03 |
| Cellular response to heat stress | 3.26E-04 | 1.17E-02 | Post NMDA receptor activation events | 1.26E-04 | 4.30E-03 |
| Nonsense Mediated Decay (NMD) independent of the Exon Junction Complex (EJC) | 3.47E-04 | 1.18E-02 | Platelet calcium homeostasis | 1.53E-04 | 4.88E-03 |
| Platelet degranulation | 3.67E-04 | 1.21E-02 | Activation of NMDA receptors and postsynaptic events | 1.74E-04 | 5.14E-03 |
| Cellular responses to stress | 4.20E-04 | 1.30E-02 | mitochondrial fatty acid beta-oxidation of unsaturated fatty acids | 1.77E-04 | 5.14E-03 |
| Viral mRNA Translation | 4.68E-04 | 1.37E-02 | Transport of small molecules | 1.92E-04 | 5.37E-03 |
| Response to elevated platelet cytosolic Ca2+ | 4.72E-04 | 1.37E-02 | Protein-protein interactions at synapses | 2.35E-04 | 6.33E-03 |
| Formation of a pool of free 40S subunits | 4.96E-04 | 1.39E-02 | Serotonin Neurotransmitter Release Cycle | 3.15E-04 | 7.89E-03 |
| Integration of energy metabolism | 8.11E-04 | 2.07E-02 | Neurotransmitter release cycle | 3.16E-04 | 7.89E-03 |
| L13a-mediated translational silencing of Ceruloplasmin expression | 8.55E-04 | 2.07E-02 | Apoptotic execution phase | 3.50E-04 | 8.39E-03 |
| Ion homeostasis | 8.69E-04 | 2.07E-02 | Ion channel transport | 4.63E-04 | 1.07E-02 |
| SRP-dependent cotranslational protein targeting to membrane | 8.99E-04 | 2.07E-02 | Unblocking of NMDA receptors, glutamate binding and activation | 5.59E-04 | 1.19E-02 |
| GTP hydrolysis and joining of the 60S ribosomal subunit | 8.99E-04 | 2.07E-02 | Negative regulation of NMDA receptor-mediated neuronal transmission | 5.59E-04 | 1.19E-02 |
| Post NMDA receptor activation events | 9.61E-04 | 2.07E-02 | HSP90 chaperone cycle for steroid hormone receptors (SHR) | 5.64E-04 | 1.19E-02 |
| Influenza Viral RNA Transcription and Replication | 1.07E-03 | 2.07E-02 | Apoptotic cleavage of cellular proteins | 6.10E-04 | 1.22E-02 |
| Nonsense-Mediated Decay (NMD) | 1.10E-03 | 2.07E-02 | Detoxification of Reactive Oxygen Species | 6.85E-04 | 1.37E-02 |

| | | | | | |
|---|---|---|---|---|---|
| Nonsense Mediated Decay (NMD) enhanced by the Exon Junction Complex (EJC) | 1.10E-03 | 2.07E-02 | Dopamine Neurotransmitter Release Cycle | 7.83E-04 | 1.49E-02 |
| p130Cas linkage to MAPK signaling for integrins | 1.15E-03 | 2.07E-02 | Branched-chain amino acid catabolism | 7.83E-04 | 1.49E-02 |
| GRB2:SOS provides linkage to MAPK signaling for Integrins | 1.15E-03 | 2.07E-02 | Glutamate Neurotransmitter Release Cycle | 9.16E-04 | 1.61E-02 |
| Activation of NMDA receptors and postsynaptic events | 1.22E-03 | 2.16E-02 | HSF1-dependent transactivation | 9.16E-04 | 1.61E-02 |
| Eukaryotic Translation Initiation | 1.27E-03 | 2.16E-02 | Cellular responses to stress | 9.48E-04 | 1.61E-02 |
| Cap-dependent Translation Initiation | 1.27E-03 | 2.16E-02 | Long-term potentiation | 1.06E-03 | 1.77E-02 |
| L1CAM interactions | 1.33E-03 | 2.21E-02 | Signaling by BRAF and RAF fusions | 1.11E-03 | 1.77E-02 |
| Ras activation upon Ca2+ influx through NMDA receptor | 1.38E-03 | 2.21E-02 | Signaling by moderate kinase activity BRAF mutants | 1.29E-03 | 1.87E-02 |
| Protein-protein interactions at synapses | 1.53E-03 | 2.35E-02 | Paradoxical activation of RAF signaling by kinase inactive BRAF | 1.29E-03 | 1.87E-02 |
| Metabolism of amino acids and derivatives | 1.57E-03 | 2.35E-02 | Reversible hydration of carbon dioxide | 1.31E-03 | 1.87E-02 |
| Formation of Fibrin Clot (Clotting Cascade) | 2.00E-03 | 3.00E-02 | Beta oxidation of myristoyl-CoA to lauroyl-CoA | 1.33E-03 | 1.87E-02 |
| Translation | 2.18E-03 | 3.26E-02 | Beta oxidation of palmitoyl-CoA to myristoyl-CoA | 1.33E-03 | 1.87E-02 |
| Cellular responses to external stimuli | 2.33E-03 | 3.26E-02 | Fatty acid metabolism | 1.38E-03 | 1.93E-02 |
| Regulation of expression of SLITs and ROBOs | 2.39E-03 | 3.35E-02 | Interleukin-12 signaling | 1.42E-03 | 1.98E-02 |
| Metabolism | 2.50E-03 | 3.50E-02 | Cellular response to heat stress | 1.58E-03 | 2.06E-02 |
| RAF/MAP kinase cascade | 2.75E-03 | 3.85E-02 | Uptake and actions of bacterial toxins | 1.70E-03 | 2.22E-02 |
| Synaptic adhesion-like molecules | 2.97E-03 | 3.86E-02 | Platelet degranulation | 2.08E-03 | 2.71E-02 |
| Heme degradation | 3.05E-03 | 3.97E-02 | Utilization of Ketone Bodies | 2.34E-03 | 2.89E-02 |
| MAPK1/MAPK3 signaling | 3.32E-03 | 4.00E-02 | PLC beta mediated events | 2.40E-03 | 2.89E-02 |
| Signaling by moderate kinase activity BRAF mutants | 3.33E-03 | 4.00E-02 | G-protein mediated events | 2.61E-03 | 3.13E-02 |

| | | | | | |
|---|---|---|---|---|---|
| Paradoxical activation of RAF signaling by kinase inactive BRAF | 3.33E-03 | 4.00E-02 | Response to elevated platelet cytosolic Ca2+ | 2.63E-03 | 3.16E-02 |
| Listeria monocytogenes entry into host cells | 3.38E-03 | 4.06E-02 | Interleukin-12 family signaling | 3.29E-03 | 3.95E-02 |
| Common Pathway of Fibrin Clot Formation | 3.38E-03 | 4.06E-02 | Oncogenic MAPK signaling | 3.30E-03 | 3.96E-02 |
| Transmission across Chemical Synapses | 3.69E-03 | 4.18E-02 | Beta oxidation of butanoyl-CoA to acetyl-CoA | 3.62E-03 | 3.98E-02 |
| Dopamine Neurotransmitter Release Cycle | 3.83E-03 | 4.18E-02 | Signaling by RAS mutants | 3.82E-03 | 4.20E-02 |
| Nephrin family interactions | 3.83E-03 | 4.18E-02 | Glutathione conjugation | 4.34E-03 | 4.77E-02 |
| NrCAM interactions | 4.12E-03 | 4.18E-02 | Gene and protein expression by JAK-STAT signaling after Interleukin-12 stimulation | 4.34E-03 | 4.77E-02 |
| Activation of C3 and C5 | 4.12E-03 | 4.18E-02 | | | |
| Scavenging by Class F Receptors | 4.12E-03 | 4.18E-02 | | | |
| Neuronal System | 4.16E-03 | 4.18E-02 | | | |
| Innate Immune System | 4.18E-03 | 4.18E-02 | | | |
| CREB1 phosphorylation through NMDA receptor-mediated activation of RAS signaling | 4.31E-03 | 4.31E-02 | | | |

Supplementary Table 6. Full upstream regulator list for each brain region.

| | BA19 | | |
|---|---|---|---|
| **Protein** | **p-value** | **Protein** | **p-value** |
| MAPT | 6.54E-09 | C1QA | 0.0186 |
| APP | 8.85E-08 | OLIG2 | 0.0186 |
| RTN4 | 1.1E-07 | mir-181 | 0.0186 |
| PSEN1 | 2.35E-07 | mir-485 | 0.0186 |
| HTT | 2.22E-06 | KCNQ2 | 0.0186 |
| MTOR | 5.72E-06 | ANGPT2 | 0.0186 |
| ROR1 | 0.0000868 | Dst | 0.0186 |
| ROR2 | 0.0000868 | JAK2 | 0.0186 |
| Inosine | 0.000242 | NEURL1 | 0.0186 |
| LPIN1 | 0.000259 | EEF2K | 0.0186 |
| AGRN | 0.000259 | NFIC | 0.0186 |
| GALNS | 0.000259 | RAB3B | 0.0186 |
| PRNP | 0.00041 | EIF4EBP2 | 0.0186 |
| PPP3CA | 0.000422 | ST8SIA2 | 0.0186 |
| SLC6A3 | 0.000494 | QKI | 0.0186 |
| S100B | 0.000514 | melatonin | 0.0186 |
| SLC6A2 | 0.000852 | SLC16A2 | 0.0244 |
| DIO2 | 0.00094 | IL6 | 0.0244 |
| ATN1 | 0.000955 | CREBBP | 0.0261 |
| ADORA2A | 0.00126 | BSCL2 | 0.0278 |
| PPP1R15A | 0.00127 | UBA6 | 0.0278 |
| REST | 0.00158 | ST8SIA4 | 0.0278 |
| SLC30A3 | 0.00177 | PLCE1 | 0.0278 |
| MKNK1 | 0.00187 | NPTX1 | 0.0278 |
| SOD1 | 0.00225 | KRAS | 0.0278 |
| PMP22 | 0.00234 | ARRB2 | 0.0278 |
| DKK1 | 0.00234 | DCX | 0.0278 |
| GAST | 0.0027 | TP63 | 0.0278 |
| TENM1 | 0.00299 | GRM2 | 0.0278 |
| SLC18A2 | 0.00299 | GABBR2 | 0.0278 |
| ESR2 | 0.00299 | KCNIP3 | 0.0278 |
| GFAP | 0.00299 | RANBP9 | 0.0278 |
| HDAC4 | 0.00301 | HEPH | 0.0278 |
| CEBPB | 0.00372 | NCAM1 | 0.0278 |
| L-dopa | 0.00452 | MME | 0.0278 |
| Ethanol | 0.00508 | DYRK1A | 0.0278 |
| TOR1A | 0.00633 | PLD2 | 0.0278 |
| BDNF | 0.00645 | ADAM10 | 0.0288 |

| | | | |
|---|---|---|---|
| FGF1 | 0.00734 | STAT3 | 0.0299 |
| NFE2L2 | 0.00734 | beta-estradiol | 0.0305 |
| APOE | 0.00792 | JUN | 0.0338 |
| D-glucose | 0.00841 | ATP | 0.0369 |
| FMR1 | 0.00852 | SOX17 | 0.0369 |
| SIRT1 | 0.00905 | SLITRK5 | 0.0369 |
| SLC4A5 | 0.00935 | VIP | 0.0369 |
| PDCD10 | 0.00935 | SOD2 | 0.0369 |
| MYO5A | 0.00935 | ITGB1 | 0.0369 |
| GMNN | 0.00935 | mir-29 | 0.0369 |
| JAK1 | 0.00935 | TGFA | 0.0369 |
| TFAP4 | 0.00935 | FGFR3 | 0.0369 |
| SEMA3A | 0.00935 | RETN | 0.0369 |
| CX3CL1 | 0.00935 | SPTBN2 | 0.0369 |
| LDLR | 0.00935 | GRM3 | 0.0369 |
| TNR | 0.00935 | ABCA1 | 0.0369 |
| TEK | 0.00935 | SGK1 | 0.0369 |
| CDON | 0.00935 | CAMK2A | 0.0369 |
| LIFR | 0.00935 | cholesterol | 0.0369 |
| Mt3 | 0.00935 | CREB1 | 0.0417 |
| DOPEG | 0.00935 | ZNF106 | 0.0422 |
| PPARG | 0.00956 | ESR1 | 0.0422 |
| NFASC | 0.0108 | PRKCD | 0.0459 |
| LEPR | 0.012 | ZFP36L2 | 0.0459 |
| PRKN | 0.012 | NGFR | 0.0459 |
| EGR2 | 0.0125 | PKM | 0.0459 |
| TSC2 | 0.0162 | TP73 | 0.0459 |
| CPEB3 | 0.0186 | THRA | 0.0459 |
| SV2A | 0.0186 | - | - |

**Cerebellum**

| **Protein** | **p-value** | **Protein** | **p-value** |
|---|---|---|---|
| MAPT | 3.39E-15 | NAGLU | 0.012 |
| APP | 7.45E-13 | SIX5 | 0.012 |
| PSEN1 | 3.36E-12 | GPX4 | 0.012 |
| HTT | 5.17E-12 | LIFR | 0.012 |
| HDAC4 | 1.12E-11 | Mt3 | 0.012 |
| MKNK1 | 1.2E-09 | BNIP3 | 0.012 |
| BDNF | 3.18E-09 | IGF1 | 0.0146 |
| ATN1 | 2.14E-07 | PPARG | 0.0153 |
| PPP3CA | 2.86E-07 | FMR1 | 0.0165 |
| PPARGC1A | 6.29E-07 | SLC6A3 | 0.0172 |

| | | | |
|---|---|---|---|
| NTRK2 | 0.0000101 | EGF | 0.0172 |
| MTOR | 0.0000284 | ADORA2A | 0.0196 |
| RTN4 | 0.000137 | NPAS4 | 0.0238 |
| RAB3B | 0.000142 | CLASP2 | 0.0238 |
| NFE2L2 | 0.000159 | C1QA | 0.0238 |
| Sucrose | 0.000341 | OLIG2 | 0.0238 |
| REST | 0.000371 | SNCB | 0.0238 |
| SNCA | 0.000371 | CAST | 0.0238 |
| S100B | 0.000839 | ATF1 | 0.0238 |
| TRPM2 | 0.000839 | KCNQ2 | 0.0238 |
| SOD2 | 0.000839 | LCAT | 0.0238 |
| TGFA | 0.000839 | Dst | 0.0238 |
| SPTBN2 | 0.000839 | PIK3CB | 0.0238 |
| LEP | 0.000861 | NFIC | 0.0238 |
| ZFP36L2 | 0.00139 | ST8SIA2 | 0.0238 |
| LEPR | 0.00141 | melatonin | 0.0238 |
| PRNP | 0.00165 | DIO2 | 0.0257 |
| SETDB1 | 0.00191 | CTNNB1 | 0.0305 |
| NTF4 | 0.00207 | LPIN1 | 0.0354 |
| FBXO2 | 0.00207 | AGRN | 0.0354 |
| Dihydrotestosterone | 0.00249 | GALNS | 0.0354 |
| MECP2 | 0.00255 | PURA | 0.0354 |
| APOE | 0.0035 | ST8SIA4 | 0.0354 |
| SHANK3 | 0.0038 | SERPINE1 | 0.0354 |
| IL6 | 0.00398 | PLCE1 | 0.0354 |
| TSC2 | 0.00476 | NPTX1 | 0.0354 |
| TAC1 | 0.00484 | KRAS | 0.0354 |
| GFAP | 0.00484 | P2RY2 | 0.0354 |
| L-dopa | 0.00625 | ATF3 | 0.0354 |
| FGF2 | 0.00704 | KCNIP3 | 0.0354 |
| DNAJC3 | 0.00728 | RANBP9 | 0.0354 |
| SOD1 | 0.00769 | NCAM1 | 0.0354 |
| CREB1 | 0.00831 | MME | 0.0354 |
| TOR1A | 0.0102 | PLD2 | 0.0354 |
| ADAM10 | 0.0103 | NTRK3 | 0.0354 |
| FOXQ1 | 0.012 | SLC16A2 | 0.0383 |
| NFATC3 | 0.012 | CREBBP | 0.041 |
| HTR2A | 0.012 | CNTF | 0.0438 |
| MYO5A | 0.012 | STAT3 | 0.0467 |
| GMNN | 0.012 | SLCO1C1 | 0.047 |
| NRF1 | 0.012 | SLC6A4 | 0.047 |
| SYP | 0.012 | ITGB1 | 0.047 |

| | | | |
|---|---|---|---|
| TFAP4 | 0.012 | FGFR3 | 0.047 |
| CHRNA7 | 0.012 | RETN | 0.047 |
| CX3CL1 | 0.012 | ABCA1 | 0.047 |
| MARK2 | 0.012 | cholesterol | 0.047 |
| DNAJC5 | 0.012 | GAST | 0.0497 |

Supplementary Table 7. Summary of targeted MS validation analysis using one-sided Wilcoxon signed rank tests to generate SRM p-values. Directionality concordance assessed whether differential expression aligned with global proteomic findings at the peptide and protein levels. Global p-value refers to the p-value calculated from the global proteomic MS analysis for the associated protein. P-values for one peptide cannot be meaningfully determined for paired analysis.

| BA19 | | | | | | |
|---|---|---|---|---|---|---|
| Protein | Peptide | Directionality concordance | % Expression A vs C (global) | % Expression A vs C (SRM) | Global p-value | SRM p-value |
| **GFAP** | - | ✓ | 55.2 | 56 | 5.36E-06 | 0.1377 |
| | ALAAELNQLR | ✓ | - | 54 | - | 0.2188 |
| | EAASYQEALAR | ✓ | - | 60 | - | 0.3125 |
| **MAP1B** | - | ✓ | 84 | 87 | 1.86E-09 | 0.058 |
| | DLSTPGLEK | ✓ | - | 87 | - | 0.1563 |
| | PQLELIEDEEK | ✓ | - | 86 | - | 0.1875 |
| **INA** | - | ✓ | 79 | 75 | 0.001041 | 0.0088 |
| | DVDGATLAR | ✓ | - | 81 | - | 0.093 |
| | FAVFIEK | ✓ | - | 66 | - | 0.0625 |
| **GSTM3** | - | ✓ | 69 | 67 | 6.39E-04 | 0.0654 |
| | LDLDFPNLPYLLDGK | ✓ | - | 72 | - | 0.1563 |
| | LTFVDFLTYDILDQNR | ✓ | - | 65 | - | 0.1563 |
| **RNAbp** | - | ✓ | 51 | 88 | 0.000503 | 0.1973 |
| | LFVGGLSFDTNEQSLEQVFSK | ✗ | - | 117 | - | 0.406 |
| | YGQISEVVVVK | ✓ | - | 67 | - | 0.0313 |
| **CHL1** | VIAVNEVGR | ✓ | 63.6 | 83 | 0.001206241 | 0.125 |

| CB | | | | | | |
|---|---|---|---|---|---|---|
| Protein | Peptide | Directionality concordance | % Expression A vs C (global) | % Expression A vs C (SRM) | Global p-value | SRM p-value |
| **PSD3** | - | ✓ | 156 | 135 | 1.35E-08 | 0.0011 |
| | NAVSVHHALASK | ✓ | - | 141 | - | 0.0234 |
| | DHYLEFEK | ✓ | - | 130 | - | 0.0059 |
| **SYN1** | - | ✓ | 136 | 129 | 6.30E-06 | 0.021 |
| | QASQAGPVPR | ✓ | - | 129 | - | 0.0762 |
| | QTSVSGPAPPK | ✓ | - | 129 | - | 0.043 |
| **NEFM** | - | ✓ | 70 | 48 | 1.21E-05 | 0.0009 |
| | FVEEIIEETK | ✓ | - | 75 | - | 0.0352 |
| | SEMEEALTAITEELAVSMK | ✓ | - | 24 | - | 0.0059 |
| **HBD** | - | ✓ | 56.8 | 72.8 | 4.64E-11 | 0.0634 |
| | EFTPQMQAAYQK | ✓ | - | 63 | - | 0.0781 |
| | VHLTPEEK | ✓ | - | 73 | - | 0.1504 |
| **VAMP2** | - | ✓ | 173 | 121 | 0.002818185 | 0.0122 |
| | ADALQAGASQFETSAAK | ✓ | - | 149 | - | 0.0508 |
| | LQQTQAQVDEVVDIMR | ✓ | - | 117 | - | 0.1016 |
| **MAPT** | - | ✓ | 138 | 113 | 0.001067 | 0.1931 |
| | IGSLDNITHVPGGGNK | ✓ | - | 115 | - | 0.2129 |
| | SPVVSGDTSPR | ✓ | - | 105 | - | 0.3965 |

Supplementary Table 8. Cell type-specific markers in BA19 and CB. 'Yes' and 'No' indicate whether the specified protein is quantified in BA19 or CB. A single asterisk indicates $p < 0.05$ between ASD and Control from the regression model, while two astericks indicate $q < 0.05$. Proteins with multiple isoforms are denoted in parentheses.

**CELL SPECIFIC MARKERS**

| CELL TYPE | Marker | BA19 | CB |
|---|---|---|---|
| ASTROCYTES | GFAP | Yes** | Yes* |
| | S100 | Yes | Yes * |
| | SLC1A3 | Yes* | Yes |
| NEURONS | NEFH/NEFL/NEFM | Yes ( /*/**) | Yes (**/**/**) |
| | RFOX3 | No | No |
| | ELAVL4 | Yes | Yes |
| | ELAVL1 | Yes | Yes |
| | TUBB3 | Yes* | Yes |
| GABAERGIC[1,2] | GAD2 | Yes | Yes |
| | GAD1 | No | No |
| | ABAT | Yes | Yes |
| | GAT1 | No | No |
| | GABRB | Yes | Yes* |
| GLUTAMATERGIC[1,3] | SLC17A7 | Yes | Yes** |
| | SLC17A6 | No | No |
| | GLS | Yes | Yes |
| | GLUL | Yes | Yes |
| | GRIN1 | No | No |
| PURKINJE CELLS[4,5] | CBLN | No | Yes |
| | CALB | Yes* | Yes* |
| | ITPR1 | Yes | Yes** |
| PYRAMIDAL CELLS[6,7,8,9] | CAMK2(A/B/C/D) | Yes (A/B*/C/D) | Yes (A/B**/C*/D) |
| | MAP2 | Yes* | Yes |
| | DLG4 | Yes** | Yes |
| | EMX1 | No | No |
| MICROGLIA | PTPRC | No | No |
| | AIF1 | No | No |
| | CD68 | No | No |
| | CIITA | Yes | No |
| | ITGAM | No | No |
| | P2RY12 | No | No |
| OLIGODENDROCYTES | MBP | Yes | Yes |
| | PLP | Yes | Yes |
| | MOG | Yes | Yes |
| | OLIG2 | No | No |
| MAST CELLS[19] | KIT | No | No |
| | FCER1 | No | No |
| | CD2 | No | No |
| | IL2RA | No | No |
| | CR1 | No | No |
| ENDOTHELIAL CELLS[11] | F8C | No | No |
| | PECAM1 | No | No |
| | ACE | No | No |
| | THBD | No | No |
| | ATXN1 | No | No |
| | HEMCAM | No | No |
| RED BLOOD CELLS[12] | CAT | Yes | Yes** |
| | HBA/HBB/HBD | Yes | Yes (**/*/**) |

[1]The Brain-specific Proteome. The Human Protein Atlas. http://www.proteinatlas.org/humanproteome/brain
[2] GABAergic neuron markers and their functions. http://www.abcam.com/neuroscience/gabaergic-neuron-markers-and-their-functions
[3]Glutamatergic neuron markers and their functions. http://www.abcam.com/neuroscience/glutamatergic-neuron-markers-and-their-functions
[4] Fernandez-Pol, A. A novel marker for Purkinje cells, ribosomal protein MPS1/S27: expression of MPS1 in human cerebellum. *Cancer Genomics Proteomics* 13, 47-53 (2016).
[5] Slemmon, J.R. *et al*. Cerebellin: a quantifiable marker for Purkinje cell maturation. *Proc Natl Acad Sci USA* 82, 7145-7148 (1985).
[6] Muller, J.F., Mascagni, F. & McDonald, A.J. Pyramidal cells of the rat basolateral amygdala: synaptology and innervation by parvalbumin-immunoreactive interneurons. *J Comp Neurol* 494, 635-650 (2006).
[7] Hayashi, N., Oohira, A. & Miyata, S. Synaptic localization of receptor-type protein tyrosine phosphatase ζ/β in the cerebral and hippocampal neurons of adult rats. *Brain Res* 1050, 163-169 (2005).
[8] Kawachi, H. *et al*. Protein tyrosine phosphatase ζ/RPTPβ interacts with PSD-95/SAP90 family. *Molec Brain Res* 72, 47-54 (1999).
[9] Chan, C-H. *et al*. Emx1 is a marker for pyramidal neurons of the cerebral cortex. *Cereb Cortex* 11, 1191-1198 (2001).
[10] Hauswirth, A. *et al*. Expression of cell surface antigens on mast cells. Mast Cells: Methods and Protocols 77-90 (2005).
[11] Garlanda, C. & Dejana, E. Heterogeneity of endothelial cells specific markers. *Arterioscl Thromb Vasc Biol* 17, 1193-1202 (1997).
[12] You, J.S. *et al*. The impact of blood contamination on the proteome of cerebrospinal fluid. *Proteomics* 5, 290-296 (2005).

Supplementary Figure 1. Representative box plots of cell type-specific marker analysis for red blood cells (HBB), neurons (NEFM), astrocytes (GFAP), and oligodendrocytes (MBP) for matched pairs in each brain region. All paired t-test analyses were non-significant ($p > 0.05$).

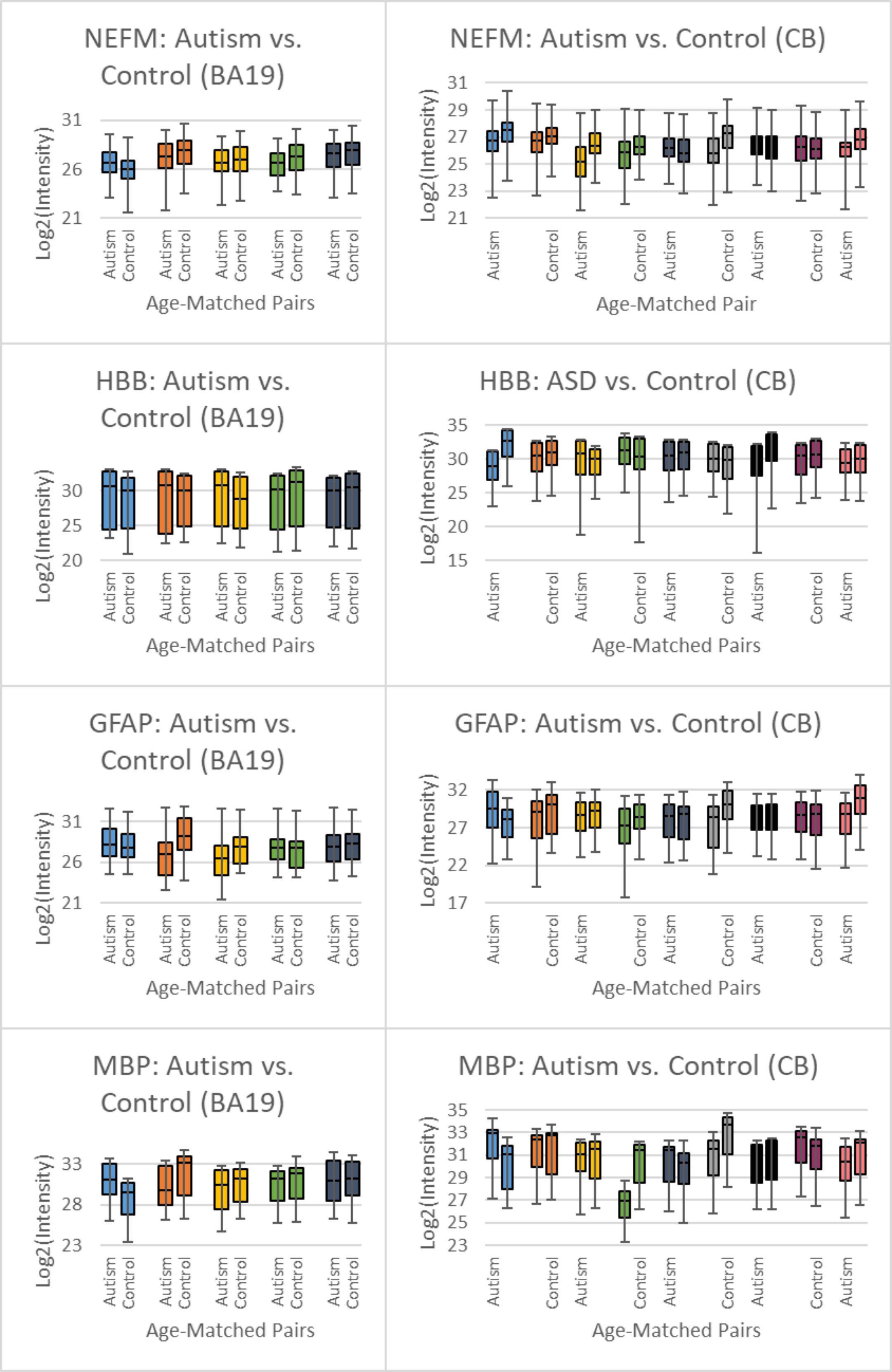

Supplement: Supplementary file 1 — Supplementary Tables and Figures [file 41598_2019_49533_MOESM1_ESM.pdf]
